# Biocontrol of strawberry gray mold caused by *Botrytis cinerea* with the termite associated *Streptomyces* sp. sdu1201 and actinomycin D

**DOI:** 10.3389/fmicb.2022.1051730

**Published:** 2022-11-04

**Authors:** Daojing Yong, Yue Li, Kai Gong, Yingying Yu, Shuai Zhao, Qiong Duan, Cailing Ren, Aiying Li, Jun Fu, Jinfeng Ni, Youming Zhang, Ruijuan Li

**Affiliations:** ^1^Helmholtz International Lab for Anti-Infectives, Shandong University-Helmholtz Institute of Biotechnology, State Key Laboratory of Microbial Technology, Shandong University, Qingdao, China; ^2^Qingdao Zhongda Agritech Co., Ltd., Qingdao, China; ^3^Chinese Academy of Sciences (CAS) Key Laboratory of Quantitative Engineering Biology, Shenzhen Institute of Synthetic Biology, Shenzhen Institute of Advanced Technology, Chinese Academy of Sciences, Shenzhen, China

**Keywords:** gray mold, biocontrol, *Streptomyces*, antifungal activity, actinomycin D

## Abstract

Strawberry gray mold caused by *Botrytis cinerea* is one of the most severe diseases in pre- and post-harvest periods. Although fungicides have been an effective way to control this disease, they can cause serious “3R” problems (Resistance, Resurgence and Residue). In this study, *Streptomyces* sp. sdu1201 isolated from the hindgut of the fungus-growing termite *Odontotermes formosanus* revealed significant antifungal activity against *B. cinerea*. Four compounds (**1**–**4**) were isolated from *Streptomyces* sp. sdu1201 and further identified as actinomycins by the HRMS and 1D NMR data. Among them, actinomycin D had the strongest inhibitory activity against *B. cinerea* with the EC_50_ value of 7.65 μg mL^−1^. The control effect of actinomycin D on strawberry gray mold was also tested on fruits and leaves *in vitro*, and its control efficiency on leaves was 78.77% at 3 d. Moreover, actinomycin D can also inhibit the polarized growth of germ tubes of *B. cinerea*. Therefore, *Streptomyces* sp. sdu1201 and actinomycin D have great potential to gray mold as biocontrol agents.

## Introduction

Strawberry (*Fragaria × ananassa* Duch.) is an important and high-value plant which is grown all over the world (Giampieri et al., 2015; Petrasch et al., 2019). Strawberry fruits are rich in sugar, dietary fiber, vitamins and amino acids and they can be processed into various by-products with high economic value (Hassan et al., 2021). However, strawberries are susceptible to microbial infections in pre- and post-harvest periods, especially fungal infection which has the greatest impact on the economic benefits of strawberry (Cantora, 2011). For example, gray mold caused by the necrotrophic fungus *Botrytis cinerea* is one of the most devastating diseases in strawberry cultivation, generally causing a 10% ~ 25% yield reduction and serious losses of more than 50% (Nguyen et al., 2015; Zhang et al., 2015; Hassan et al., 2021). Furthermore, *B. cinerea* can also infect post-harvest strawberry fruits, causing huge economic losses in the process of storage, transportation and sales (Chen et al., 2018).

Traditionally, chemical control has been the most effective strategy for strawberry gray mold control for years (de Moura et al., 2021; Fontana et al., 2021; Zhou et al., 2021). However, frequent and large-scale use of numerous fungicides leads to the increasingly serious “3R” problems (Resistance, Resurgence and Residue) (de Moura et al., 2021). Therefore, it is urgent to explore new strategies or alternatives to chemical fungicides to control strawberry gray mold.

Biocontrol of *B. cinerea* using plant extracts or biocontrol agents (BCAs) has become an availably alternative to chemical control, mainly due to its environmentally friendly and non-cross resistant (Nguyen et al., 2015; Sernaite et al., 2020; Iqbal et al., 2022). Plant extracts, for example, cinnamon extracts, clove extracts, and ajwain essential oils have been reported to control strawberry gray mold with significant effects *in vitro* (Zamani-Zadeh et al., 2013; Sernaite et al., 2020; Fontana et al., 2021). Different BCAs applied to strawberry gray mold come from fungi, bacillus and actinomycetes have different modes of action, including induced resistance, competition for nutrients, production of bioactive metabolites, and parasitism (Zhang et al., 2015; Raza et al., 2016; Spadaro and Droby, 2016). At present, *Trichoderma* and yeast are the main antagonistic fungi to control gray mold. Greenhouse studies revealed that fungal antagonists *Trichoderma koningii* (T21) can reduce the gray mold disease severity with 56% (Sharifi-Tehrani and Alizadeh, 2000; Alizadeh et al., 2007). The yeast strain *Hanseniaspora uvarum* plays an important role in biocontrol of gray mold of strawberry by suppression of conidial germination and hyphal growth of *B. cinerea*, at the same time its secondary metabolites also exhibit antifungal activity on *B. cinerea* (Qin et al., 2017). It is reported that some antagonistic bacillus can also effectively control strawberry gray mold, such as *Bacillus cereus* (Yu et al., 2021), *Bacillus subtilis* (Hang et al., 2005), *Bacillus licheniformis* (Kim et al., 2007), and *Paenibacillus polymyxa* (Tsai et al., 2022). Furthermore, actinomycetes used to control strawberry gray mold have been extensively exploited due to their ability to produce secondary metabolites that are active against *B. cinerea* (Silva et al., 2014; Kim et al., 2015; Lyu et al., 2017).

Searching new active secondary metabolites from special eco-environment microorganisms has become a research hotspot, since rediscovery of natural products from microorganisms isolated from soil has entering the bottleneck period (Seipke et al., 2012; Brune, 2014; Sujada et al., 2014; Sayed et al., 2020; Li J. et al., 2021). It is reported that there are a large number of microorganisms in the hindgut of termites, and their metabolites are an important source of carbon frame for nutrition and self-material synthesis (Hongoh, 2010; Xu et al., 2020). Therefore, underexploited termite symbionts can offer a pathway to find unreported biological resources. In our present study, an intestinal symbiont bacterium was isolated from the hindgut of the fungus-growing termite *Odontotermes formosanus*. Based on the analysis of morphological and alignment of 16S rRNA sequence, the strain was designated as *Streptomyces* sp. sdu1201. The fermentation broth of *Streptomyces* sp. sdu1201 had broad-spectrum antifungal activities against six plant pathogenic fungi, among which the best inhibitory effect was against *B. cinerea*. The main products from *Streptomyces* sp. sdu1201 were identified as actinomycins, among which actinomycin D showed significantly inhibitory activity on the growth of mycelium and the elongation of germ tubes. Furthermore, the control effect on strawberry gray mold *in vitro* was verified. These results suggested that *Streptomyces* sp. sdu1201 and actinomycin D had great potential for development and application as BCAs in the control of strawberry gray mold.

## Materials and methods

### Strains and culture conditions

*Streptomyces* sp. sdu1201 was isolated from the hindgut of the fungus-growing termite *O. formosanus* with the method described by Li J. et al. (2021), and termite nests were collected in Shaoguan (113.59°E, 24.82°N), Guangdong province, China in June 2018. It is cultured on mannitol soybean agar (MS, 20 g of soybean, 20 g of mannitol and 12 g of agar in 1000 ml distilled water) plates at 30°C, and six pathogenic fungi (*Fusarium graminearum*, *Botryosphaeria dothidea*, *Fusarium oxysporum*, *Botrytis cinerea*, *Glomerella cingulate* and *Alternaria solani* are phytopathogen responsible for wheat scab, apple ring rot, cotton Fusarium wilt, strawberry gray mold, Glomerellaleaf spot and tomato early blight, respectively) are cultured on potato dextrose agar (PDA, the extract of 200 g of potato, 20 g of glucose and 12 g of agar in 1000 ml distilled water) at 25°C. All strains are stored in the lab of Shandong University–Helmholtz Institute of Biotechnology.

### Identification and whole-genome sequencing of *Streptomyces* sp. sdu1201

Genomic DNA of the strain sdu1201 was extracted according to the protocol in our labs (Wang et al., 2016). Polymerase chain reaction (PCR) amplification of the 16S rRNA gene was performed using the universal primers of 27F (5′-AGAGTTTGATCMTGGCTCAG-3′) and 1492R (5′-GGTTACCTTGTTACGACTT-3′) with PrimeSTAR® Max DNA Polymerase (TaKaRa), and its product was sequenced by RuiBiotech (Qingdao, China). The 16S rRNA gene sequences were compared using the BLAST analysis, and the multiple sequence alignment based on CLUSTALW. The phylogenetic tree was constructed with the maximum-likelihood method in MEGA X.

The whole genome of *Streptomyces* sp. sdu1201 was sequenced by Biomarker Technologies Co., Ltd. (Qingdao China). Analysis and annotation of biosynthetic gene clusters (BGCs) of secondary metabolites were done with online antiSMASH 6.0 version. Comparison of BGCs were showed using Easyfig 2.2.5 as the visualization tool (Sullivan et al., 2011).

### Screening of fermentation medium

*Streptomyces* sp. sdu1201 was grown on MS plates at 30°C for 7 days. A single colony was inoculated in tryptic soy broth (TSB, 17 g of casein tryptone, 3 g of enzymatic digest of soybean meal, 5 g of NaCl, 2.5 g of K_2_HPO_4_ and 2.5 g of glucose in 1000 ml distilled water, pH 7.3) of 50 ml/250 ml conical flask, with shaking culture (200 rpm) at 30°C for 30 h, which is used as seed broth. Seed broth was transferred in fermentation media (50 ml/250 ml conical flask) with 2% (v/v) of inoculation volume, and then incubated at 30°C for 8 days on a rotary shaker at 200 rpm. The fermentation broth was adsorbed by amberlite XAD-16 resin (1 ml/50 ml fermentation broth) for 12 h, and centrifuged, and precipitates was extracted with 30 ml methanol for 4 h. After filtration, extracts were evaporated to dryness under reduced pressure by a rotary evaporator. Crude extracts were redissolved with 1 ml MeOH, its chemo-diversity was investigated by HPLC-MS. Parameters of HPLC and MS were the same as described by Li R. et al. (2021).

Five different fermentation media were used: (a) SSM medium consisting of glucose 1%, (NH_4_)_2_SO_4_ 0.2%, NaCl 0.2%, KH_2_PO_4_ 0.05%, K_2_HPO_4_ 0.1%, MgSO_4_ 0.2%, CaCO_3_ 0.5% and yeast extract 0.2% (pH 7.0); (b) ISP-2 (International *Streptomyces* Project 2) medium consisting of yeast extract 0.4%, malt extract powder 1% and glucose 0.4% (pH 7.2); (c) ISP-4 (International *Streptomyces* Project 4) medium consisting of soluble starch 1%, K_2_HPO_4_ 0.1%, MgSO_4_·7H_2_O 0.1%, NaCl 0.1%, (NH_4_)_2_SO_4_ 0.2%, CaCO_3_ 0.2%, FeSO_4_·7H_2_O 0.0001% and MnCl_2_·4H_2_O 0.0001% (pH 7.0 ~ 7.4); (d) ISP-7 (International *Streptomyces* Project 7) medium consisting of glycerol 1.5%, L-tyrosine 0.05%, L-asparagine 0.1%, K_2_HPO_4_ 0.05%, MgSO_4_·7H_2_O 0.05%, NaCl 0.05%, FeSO_4_·7H_2_O 0.001% and trace salts solution 0.1% (FeSO_4_·7H_2_O 0.1%, MnCl_2_·4H_2_O 0.1%, ZnSO_4_·7H_2_O 0.1%) (pH 7.2 ~ 7.4); (e) R5A medium consisting of sucrose 10%, glucose 1%, yeast extract 0.5%, MgCl_2_·6H_2_O 1%, K_2_SO_4_ 0.025%, casamino acids 0.01%, MOPS 2.1%, NaOH 0.2%, CaCl_2_ 5.88 mg L^−1^, ZnCl_2_ 80 μg L^−1^, FeCl_3_·6H_2_O 400 μg L^−1^, MnCl_2_ 20 μg L^−1^, CuCl_2_ 20 μg L^−1^, Na_2_B_4_O_7_·10H_2_O 20 μg L^−1^ and (NH_4_)_6_Mo_7_O_24_·4H_2_O 20 μg L^−1^ (pH 6.85).

### Antifungal activity assay of *Streptomyces* sp. sdu1201

The inhibitory effects of *Streptomyces* sp. sdu1201 on six pathogenic fungi were determined with the method of mycelium growth rate (Rong et al., 2020; Cimen et al., 2021). Seed broth of *Streptomyces* sp. 1201 was cultured in ISP-7 (50 ml/250 ml conical flask) at 30°C for 8 days on a rotary shaker at 200 rpm with 2% (v/v) of inoculation volume. The fermentation broth was added to Luria-Bertani (LB) medium (4% v/v) which was precooled to about 50°C, and the solution was mixed thoroughly before pouring the plates. The LB plates without fermentation broth were used as controls. Six millimeter-diameter disks of pathogenic fungi were inoculated on these plates, which were cultured at 25°C for about 3 d with darkness. The mycelium diameter was measured by cross method. Three replicates were set for each treatment.


$$\mathrm{Inhibition rate}=\frac{\mathrm{mycelium diameter in control}-\mathrm{mycelium diameter in treatment}}{\mathrm{mycelium diameter in control}-\mathrm{disk diameter}}\times 100\%$$

Antifungal activity of the supernatant and cell culture against *B. cinerea* were done with the same method. The supernatant was collected by centrifugation (8,000 × *g*, 10 min), and the precipitates were resuspended with an equivalent amount of sterile water, using as cell culture.

### Isolation and purification of compounds from *Streptomyces* sp. sdu1201

Forty liters fermentation broth was prepared to extract, isolate, and purify active compounds. Crude extracts (36 g) were collected with the method described in “Screening of fermentation medium.” The crude extracts were separated into three fractions (Frs1 ~ 3) by silica gel column chromatography, using a step gradient elution with 50:1, 30:1, 10:1 (v/v) of CH_2_Cl_2_-MeOH. Frs1 was purified by semi-preparative HPLC (ODS; Bruker ZOR-BAX SB-C18, 5 μm, 250 × 10 mm, MeOH-H_2_O, 92:8, 2 ml/min) to give **1** (61.9 mg, *t*_R_ = 10.5 min) and **2** (93.8 mg, *t*_R_ = 11.2 min). **3** (49.1 mg, *t*_R_ = 9.7 min) was purified from Frs2 with the same conditions. Frs3 was purified by semi-preparative HPLC (MeOH-H_2_O, 90:10, 2 ml/min) to yield **4** (53.1 mg, *t*_R_ = 10.1 min).

### Antifungal activity assay of compounds

Similarly, EC_50_ (effective concentration at half maximum) of compounds to *B. cinerea* was measured by mycelium growth rate method (Cimen et al., 2021). Compounds **1**–**4** were dissolved in DMSO, and diluted into a series of concentrations with deionized water containing 0.001% tween-80. The diluted compound **1** and **2** were diluted in LB medium to the final concentrations of 125, 62.5, 31.25, 15.625, 7.8125 μg mL^−1^. The final concentrations of plates prepared with the other two compounds are 1,000, 500, 250, 125, 61.25 μg mL^−1^. Equivalent DMSO (5%, v/v) was used as the control. Disks (6 mm in diameter) of *B. cinerea* were placed in the center of these plates. Measure mycelium diameter, after inoculation at 25°C for 3 d. The EC_50_ values of compounds were calculated through the linear regression analysis between the inhibition rate and the logarithm of the drug concentration (Zhang et al., 2020; Jiang et al., 2021).

### Control effect of fermentation broth of *Streptomyces* sp. sdu1201 on gray mold of postharvest strawberry

Strawberry fruits (“Tian Bao”) were chosen with the same commercial maturity, color and size, without diseases and mechanical damage. All the fruits were cleaned with running water, surface sterilized using 75% ethanol, and air-dried in clean bench. Half fruits were soaked in fermentation broth for 3 min, the others were treated with sterile water for 3 min. After 24 h, fruits were treated as following: (i) Control, fruits only treated with sterile water; (ii) S1201, fruits only treated with fermentation broth; (iii) *B. cinerea*, fruits treated with sterile water were inoculated with disks of *B. cinerea* after wounded at the equator. *B. cinerea* was cultured on PDA for 3 days, and then disks were taken with a diameter of 6 mm at the edge of the colony for standby; (iv) S1201 + *B. cinerea*, fruits treated with fermentation broth were inoculated with disks of *B. cinerea* after wounded at the equator. All strawberries were placed in an incubator at 16°C with 90% ~ 95% relative humidity for 4 days. The lesion diameter in all the treatments were measured at 1 d, 2 d, 3 d and 4 d to calculate lesion area and control efficiency, and then disease severity were recorded by taking photos at the same time. The experiment was repeated for three times.


$$\mathrm{Control efficiency}=\frac{\mathrm{lesion area in}\;B.cinerea-\mathrm{lesion area in}\;S1201+B.cinerea}{\mathrm{lesion area in}\;B.cinerea}\times 100\%$$

### Protective effects of compound 2 against *Botrytis cinerea* on strawberry leaves

The second and third strawberry leaves (“Tian Bao”) with uniform size, without diseases and mechanical damage at the tip were selected to test protective effects of compound **2**. The leaves were treated in the same way described by Wang et al. (Wang et al., 2014). Half of leaves were sprayed with **2** (62.5 μg mL^−1^) until drops fall, and the other was treated with sterile water. All leaves were treated as follows immediately after air-drying: (i) Control, leaves treated with sterile water; (ii) Compound **2**, leaves treated with compound **2**; (iii) *B. cinerea*, leaves treated with sterile water were inoculated with *B. cinerea* after wounded (four for each leave), *B. cinerea* was cultured on PDA for 3 days, and then disks were taken with a diameter of 6 mm at the edge of the colony for standby; (iv) Compound **2** + *B. cinerea*, leaves treated with compound **2** were inoculated with *B. cinerea* after wounded (four for each leave). After that, the leaves were transferred to sterile plastic boxes with covers, and incubated for 4 days at 25°C with about 95% relative humidity. The incidence of leaves was recorded every day, and control efficiency was calculated with the above formula. The experiment was repeated for three times.

### Inhibitory effect of compound 2 against germ tube elongation of *Botrytis cinerea*

The inhibitory effect of compound **2** on germ tube elongation of *B. cinerea* was determined by concave slide method (Sarven et al., 2020; Zhao et al., 2021). Spores of *B. cinerea* were collected from strawberry leaves after incubated 5 ~ 6 d, transfer spores to 1% glucose solution (pH 5.0), mix evenly and prepare spore suspension of 6 × 10^5^ spores mL^−1^. Compound **2** was diluted with deionized water (0.001% Tween-80) to a series of concentrations, such as 4, 2, 1, 0.5, 0.25 mg mL^−1^. After mixing 100 μl spore suspension with the same amount of compound **2** of different concentrations, 60 μl mixture were added on concave slides. Sterile water was used as a control. After then, concave slides were transferred to petri dish (120 mm in diameter) with high relative humidity, and incubated at 22°C for 24 h. Cover the petri dish with freshly soaked toilet paper to control high relative humidity. Morphology of conidia and germ tubes were taken photos using MShot Image Analysis Syst at 6 h, 12 h and 24 h and the length of germ tube were obtained by image measurement. This experiment was performed twice with three replications.

### Statistical analysis

Data analysis was performed with IBM SPSS Statistics 26.0 (SPSS Inc., Chicago, Illinois). The results were performed with ANOVA followed by Duncan’s multiple range test. A *p*-values <0.05 were considered to be statistically significant.

## Results

### Identification of the strain sdu1201

**Figure 1:**
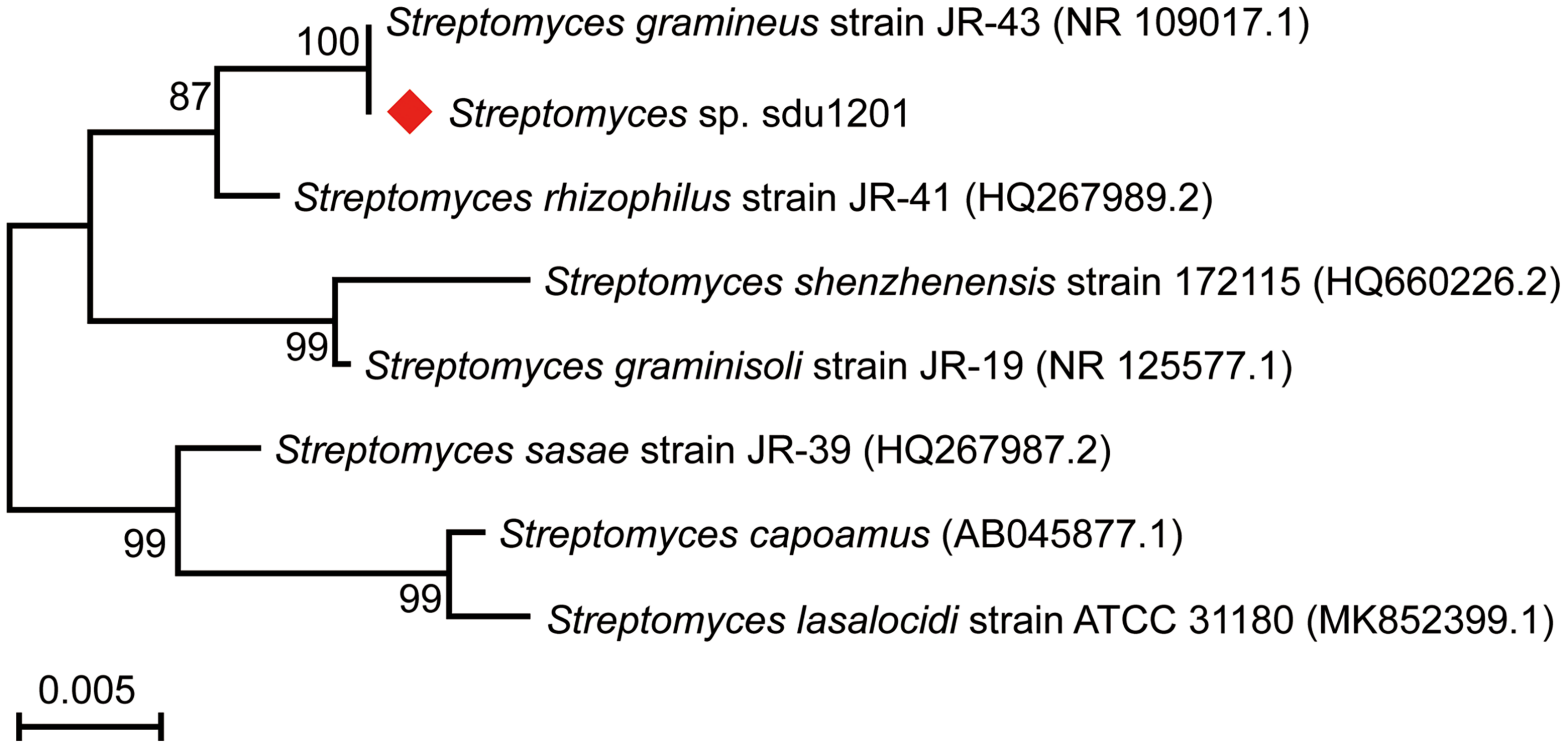
Phylogenetic tree of strain *Streptomyces* sp. sdu1201 based on 16S rRNA sequences by maximum-likelihood method.

The 16S rRNA sequence of 1,397 bp (GenBank accession No. OP432474) was amplified, sequenced, and then searched in the NCBI BLAST database. The results showed that the strain sdu1201 had high sequence identity (> 97%) with *Streptomyces*. The phylogenetic tree revealed that the strain sdu1201 and *Streptomyces gramineus* JR-43 were located in the same branch with 100% similarity (Figure 1). On the neighbor branch, *Streptomyces rhizophilus* JR-41 presented 99.07% similarity with the strain sdu1201. Moreover, the other five strains clustered on other branches showed 97.42% ~ 98.42% similarity with the strain sdu1201. Thus, the strain sdu1201 belonged to the genus of *Streptomyces* and named *Streptomyces* sp. sdu1201.

### Screening for fermentation medium of *Streptomyces* sp. sdu1201

**Figure 2:**
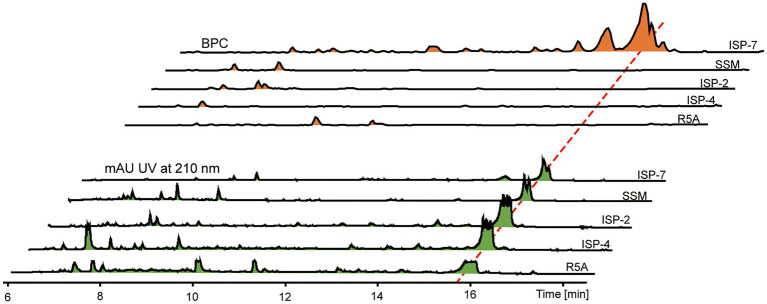
HPLC-MS data of crude extracts of *Streptomyces* sp. sdu1201 cultured in different media.

Crude extracts of *Streptomyces* sp. sdu1201 cultured in different media were determined by HPLC-MS analysis (Figure 2). There is a maximum absorption peak (15 ~ 16 min) at 210 nm for all five fermentation media. It can be seen from BPC spectra that different peak time and peak area were detected by different treatments, indicating that the types and yields of compounds produced by different fermentation media were also different. Some differential peaks were detected at 7 ~ 12 min in ISP-2, ISP-4, R5A and SSM medium. However, in ISP-7 medium, a number of differential peaks were produced at 15 ~ 17 min, with larger peak area, indicating that the crude extracts produced by ISP-7 medium are rich in variety and high in yield. Therefore, ISP-7 medium was selected as the fermentation medium of *Streptomyces* sp. sdu1201 for subsequent experiments.

### Antifungal activity of *Streptomyces* sp. sdu1201

**Figure 3:**
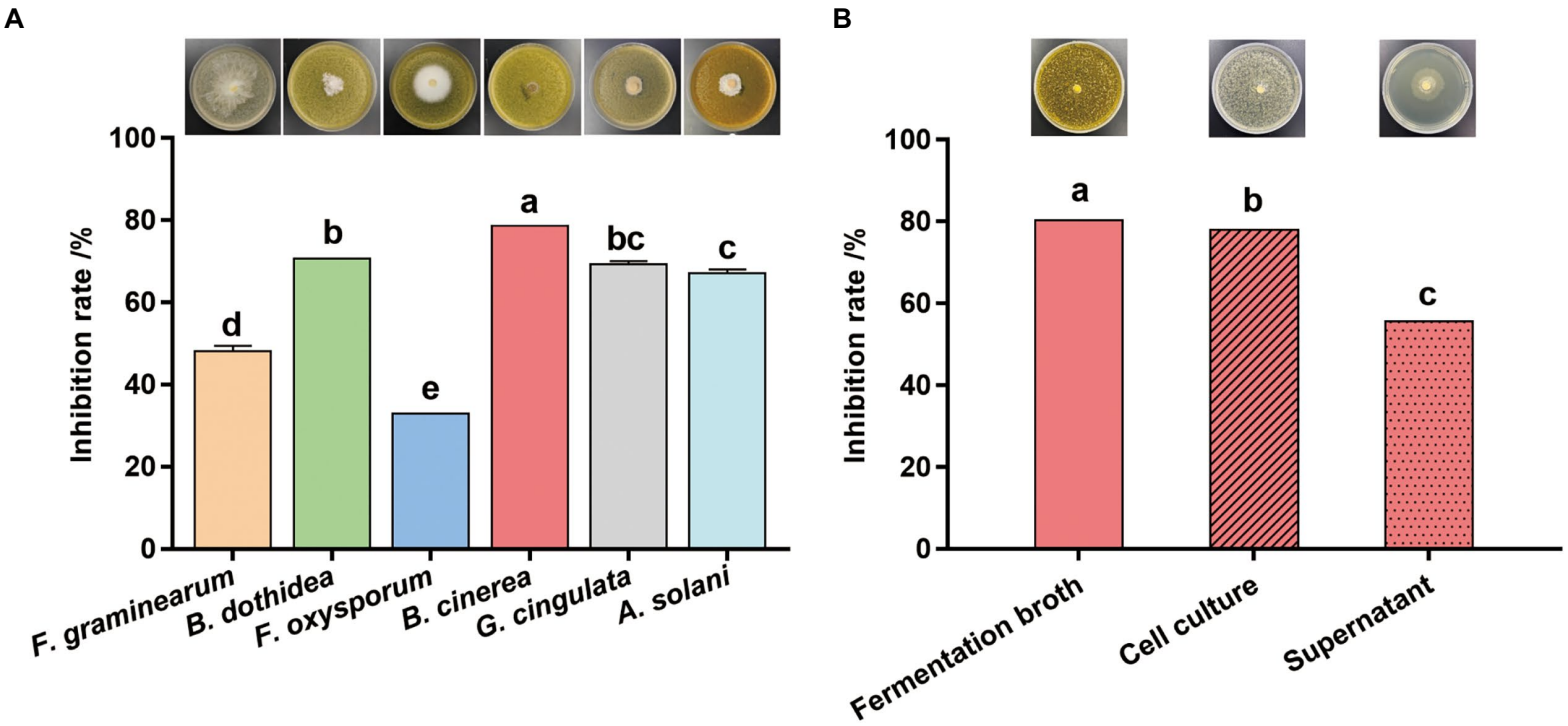
Antifungal activity of *Streptomyces* sp. sdu1201. **(A)** Antifungal activity of the fermentation broth of *Streptomyces* sp. sdu1201 against six pathogenic fungi. **(B)** The inhibitory rate of the cell culture and supernatant of *Streptomyces* sp. sdu1201 against *B. cinerea*. Vertical bars represent the standard errors of the means. Different letters above the bars indicate statistically significant differences at *p* < 0.05.

The fermentation broth of *Streptomyces* sp. sdu1201 has significant inhibitory effects against six pathogenic fungi (Figure 3A). The inhibition rate of fermentation broth to *B. cinerea* was 78.69%, which was significantly higher than that of the other five pathogenic fungi. However, *Streptomyces* sp. sdu1201 has the lowest effect on *F. oxysporum*, and the inhibitory rate is only 32.84%. There was no significant difference in antifungal activity against *B. dothidea* and *G. cingulate*, with inhibitory rate were 70.59 and 69.38%, respectively. As shown in Figure 3B, the inhibitory rate of the cell culture and supernatant against *B. cinerea* was 78.05 and 55.69%, respectively.

### Isolation and identification of actinomycins from *Streptomyces* sp. sdu1201

**Figure 4:**
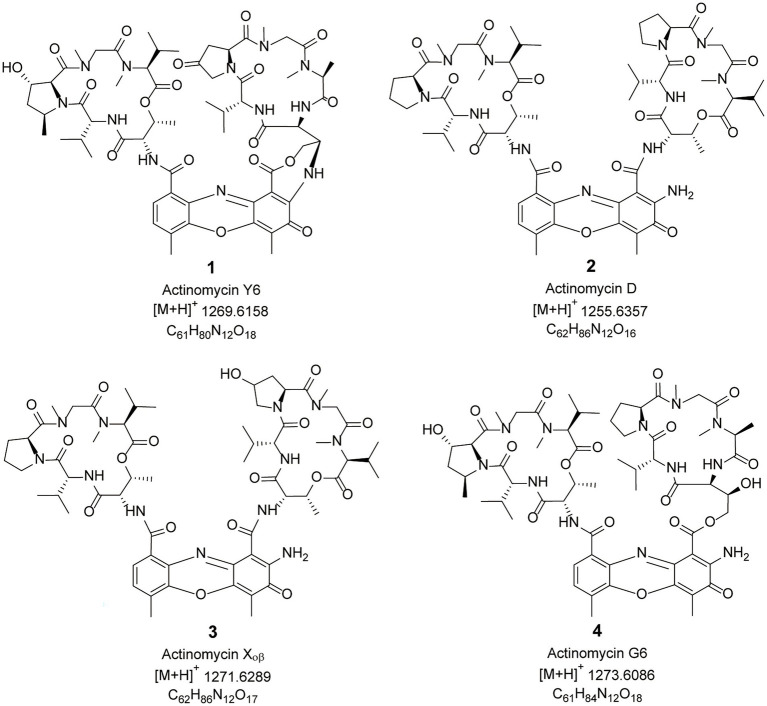
Chemical structures of compounds **1**–**4**.

Four compounds were isolated from crude extracts of *Streptomyces* sp. sdu1201 using chromatography methods. Their structures were analyzed by the techniques of HRMS and 1D NMR (Supplementary Figure 1; Supplementary Table 1). Compounds **1**–**4** (Figure 4) were orange solid, and their molecular formula were characterized as C_61_H_80_N_12_O_18_, C_62_H_86_N_12_O_16_, C_62_H_86_N_12_O_17_ and C_61_H_84_N_12_O_18_ according to the HRESIMS peaks at *m/z* 1269.6158 [M + H]^+^, 1255.6357 [M + H]^+^, 1271.6289 [M + H]^+^, 1273.6086 [M + H]^+^, respectively. Finally, compounds **1**–**4** were identified as actinomycin Y6 (Cai et al., 2016), actinomycin D (Liu et al., 2020), actinomycin X_0β_ (Wang et al., 2017), and actinomycin G6 (Jens Bitzer and Zeeck, 2006) by 1D NMR data (Supplementary Table 1; Supplementary Figures 2–5).

### Identification of the actinomycin D BGC

**Figure 5:**
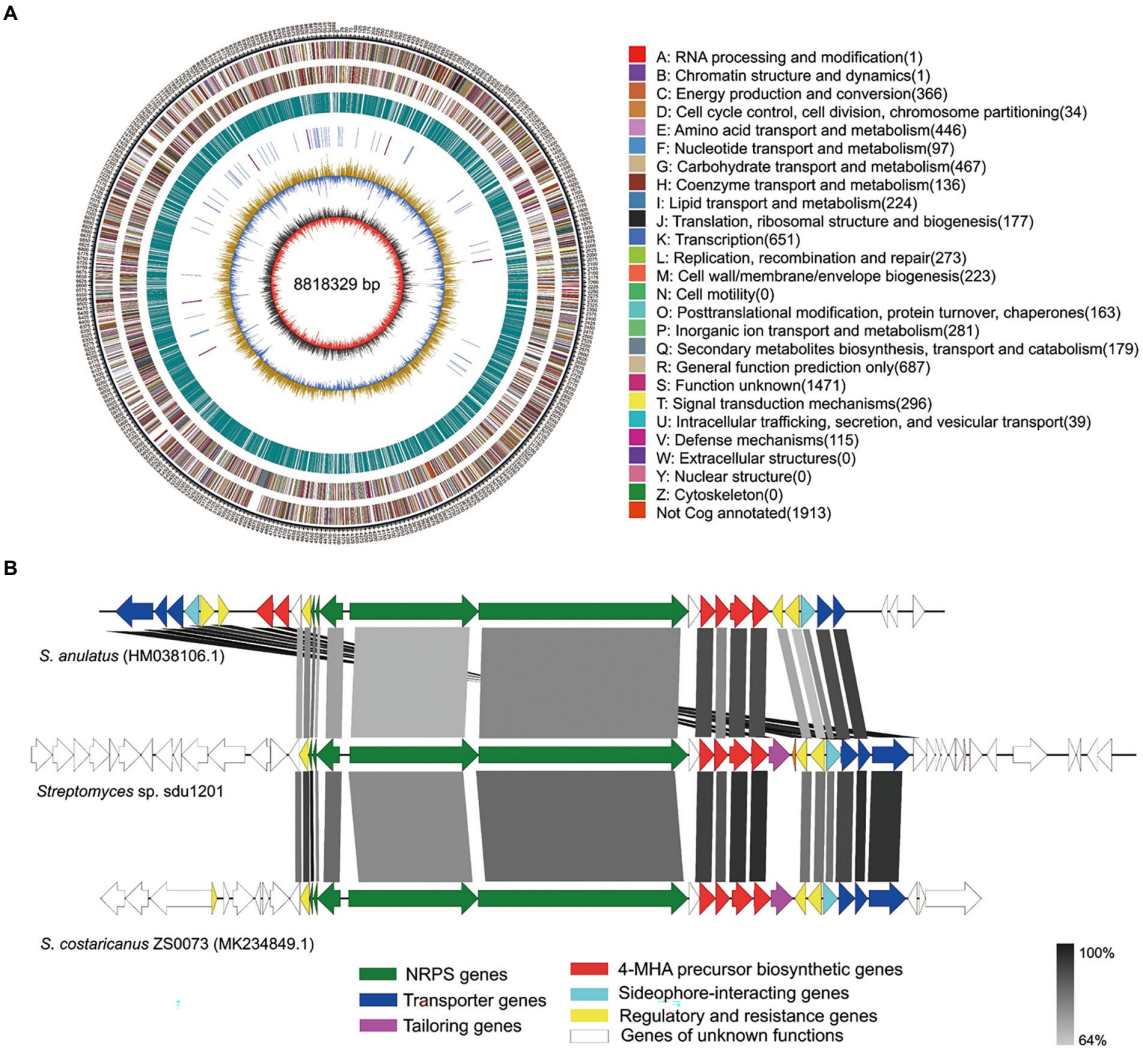
Complete genome and actinomycin D BGC of *Streptomyces* sp. sdu1201. **(A)** Circular map of genome. The seven circles (outer to inner) represent forward genome size, strand coding sequences, reverse-strand coding sequences, repetitive sequences, transfer RNA (blue) and ribosomal RNA (purple), GC content, and GC skew. **(B)** Comparison of actinomycin D BGC from *Streptomyces* sp. sdu1201 (middle) with reference actinomycin D BGC from *S. anulatus* (top) and *S. costaricanus* ZS0073 (bottom).

To identify the BGC of actinomycin D, the genome of *Streptomyces* sp. sdu1201 was sequenced. The size of complete genome of *Streptomyces* sp. sdu1201was 8,818,329 bp (Figure 5A) and 34 BGCs were predicted by antiSMASH. Among them, a NRPS gene cluster (67,075 bp) was suspected of coding for the biosynthesis of actinomycin D (GenBank accession No. OP355585). In Figure 5B, comparison of actinomycin D BGC from *Streptomyces* sp. sdu1201 with that from *S. anulatus* (HM038106.1) and *S. costaricanus* ZS0073 (MK234849.1) revealed that these three BGCs are highly similar. The size of these core NRPS genes were roughly the same, and the identity was 68.74% ~ 87.88% (Supplementary Table 2). The biosynthesis of 4-MHA was different in actinomycin-producing strains, but the homology similarity of 4-MHA BGC in *Streptomyces* sp. sdu1201 and *S. costaricanus* ZS0073 were quite high, with the identity of 80.86% ~ 87.57% (Supplementary Table 2).

### Antifungal activity against *Botrytis cinerea* of compounds 1–4

To test the antifungal activity of the isolated compounds, EC_50_ values for inhibition of *B. cinerea* were obtained (Table 1). The results showed that compound **2** had the best antifungal activity against *B. cinerea*, and its EC_50_ value was 7.65 μg mL^−1^. Compound **1** had inhibitory effect with EC_50_ value of 37.70 μg mL^−1^. The EC_50_ value of compounds **3** and **4** were 608.42 μg mL^−1^ and 535.06 μg mL^−1^, suggesting that they had no obvious inhibitory effect on *B. cinerea*. The correlation coefficients of compounds **1**–**4** ranged from 0.9849 to 0.9981, indicating that the antifungal effects of these compounds were positively correlated with its concentrations.

**Table 1:** Antifungal activity against *B. cinerea* of compounds 1–4.

Compounds	Regression equation	Correlation coefficient (R^2^)	EC_50_ (μg mL^−1^)
**1**	*y* = 2.3896x + 1.2332	0.9973	37.70
**2**	*y* = 2.3892x + 2.8886	0.9849	7.65
**3**	*y* = 5.4221x – 10.096	0.9981	608.42
**4**	*y* = 5.0995x – 8.9133	0.9940	535.06

### Bioassay against *Botrytis cinerea* on detached strawberry fruits and leaves

**Figure 6:**
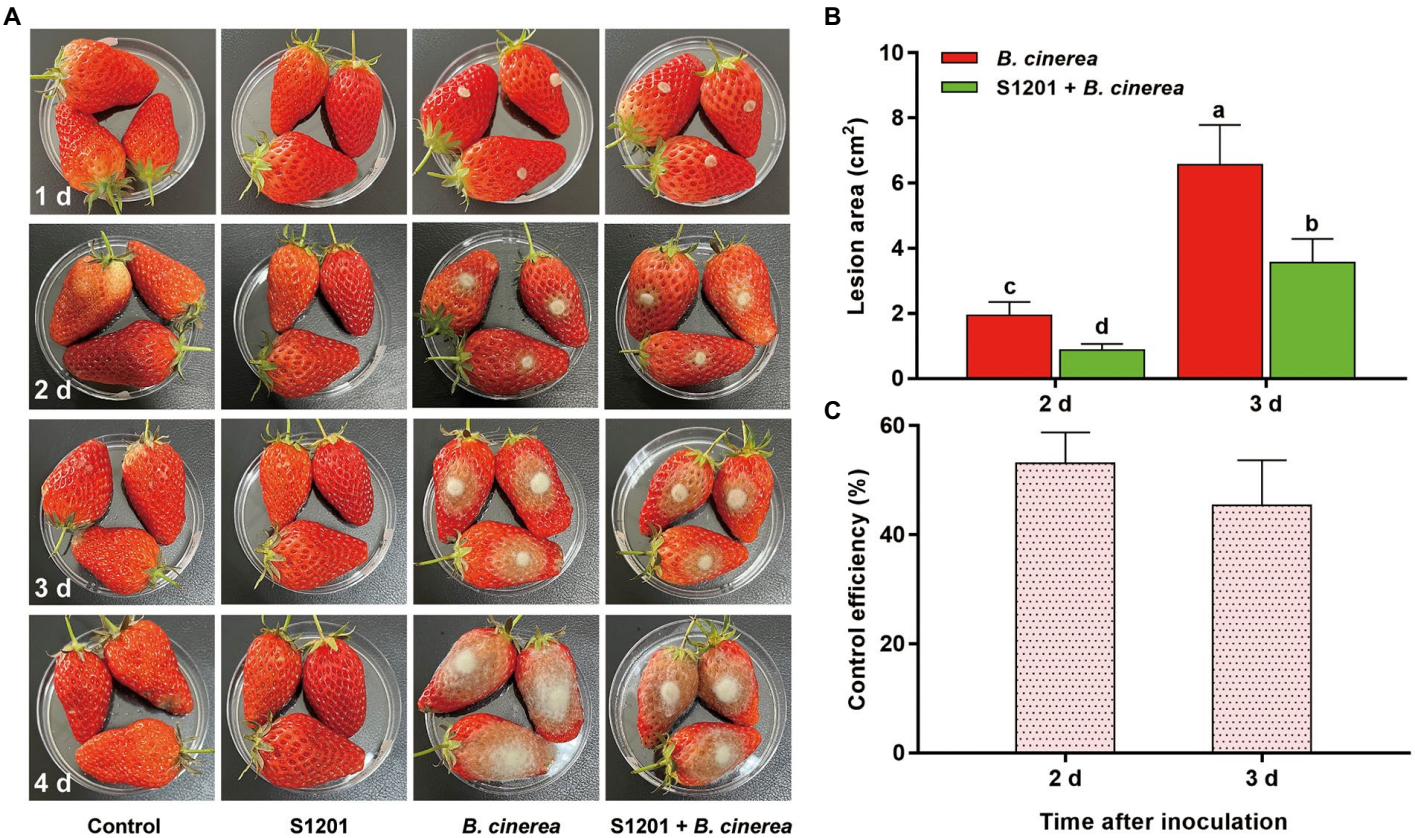
Bioassay of fermentation broth against *B. cinerea* on detached strawberry fruits. **(A)** Symptoms on strawberry fruits in different treatments. **(B)** Lesion area on strawberry fruits in *B. cinerea* and S1201 + *B. cinerea* treatment at 2 d and 3 d. **(C)** Control efficiency of fermentation broth against *B. cinerea* at 2 d and 3 d. Vertical bars represent the standard errors of the means. Different letters above the bars indicate statistically significant differences at *p* < 0.05.

The control effect of fermentation broth of *Streptomyces* sp. sdu1201 against gray mold was tested on strawberry fruits. There were no obvious symptoms in the groups of control and S1201 in 4 days (Figure 6A). However, in *B. cinerea* group, obvious symptoms can be observed at 2 d. At 3 d, the lesion area was 6.57 ± 1.22 cm^2^, and the lesion area expands rapidly with the time. In the group of S1201 + *B. cinerea*, the lesion area were 0.88 ± 0.18 cm^2^ and 3.56 ± 0.73 cm^2^ at 2 d and 3 d, respectively, which were significantly (*p* < 0.05) smaller than that in *B. cinerea* group (Figure 6B). The control efficiency of fermentation broth was 53.33 and 45.44% at 2 d and 3 d, respectively (Figure 6C).

**Figure 7:**
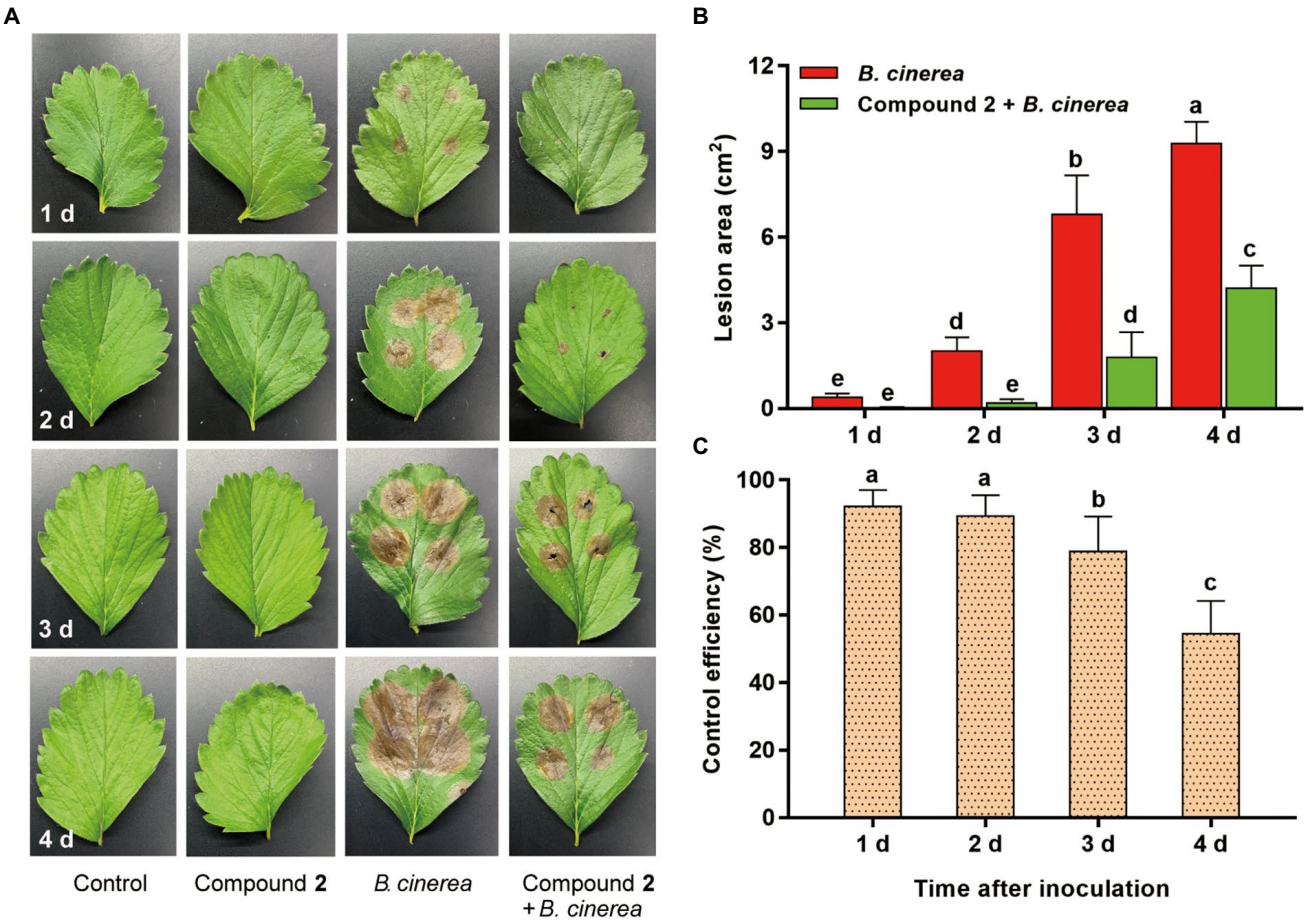
Bioassay of compound **2** against *B. cinerea* on detached strawberry leaves. **(A)** Symptoms on strawberry leaves in different treatments. **(B)** Lesion area on strawberry leaves in *B. cinerea* and compound **2** + *B. cinerea* treatment from 1 d to 4 d. **(C)** Control efficiency of compound **2** against *B. cinerea* ranging from 1 d to 4 d. Vertical bars represent the standard errors of the means. Different letters above the bars indicate statistically significant differences at *p* < 0.05.

The control efficiency of compound **2** to *B. cinerea* on detached strawberry leaves was also tested. No disease spots were observed on leaves treated with sterile water and compound **2** along with the time (Figure 7A). The lesion area in compound **2** + *B. cinerea* treatment were significantly (*p* < 0.05) smaller than those in *B. cinerea* treatment until 4 d. Obvious brown spots can be seen at 1 d after treated with *B. cinerea*, however, slight wound can be observed at 2 d in compound **2** + *B. cinerea* treatment. The lesion area in compound **2** + *B. cinerea* treatment were 1.79 ± 0.8 cm^2^ and 4.20 ± 0.80 cm^2^ at 3 d and 4 d, respectively, significantly smaller than that in the *B. cinerea* treatment, which were 6.79 ± 1.37 cm^2^ and 9.29 ± 0.76 cm^2^ at 3 d and 4 d, respectively (Figure 7B). Compound **2** had dramatically control effect of gray mold on strawberry leaves (Figure 7C), and the control efficiency was 92.22% (at 1 d), 89.39% (at 2 d), 78.77% (at 3 d), and 54.49% (at 4 d), respectively.

### Inhibitory effect of compound 2 against germ tube elongation of *Botrytis cinerea*

**Figure 8:**
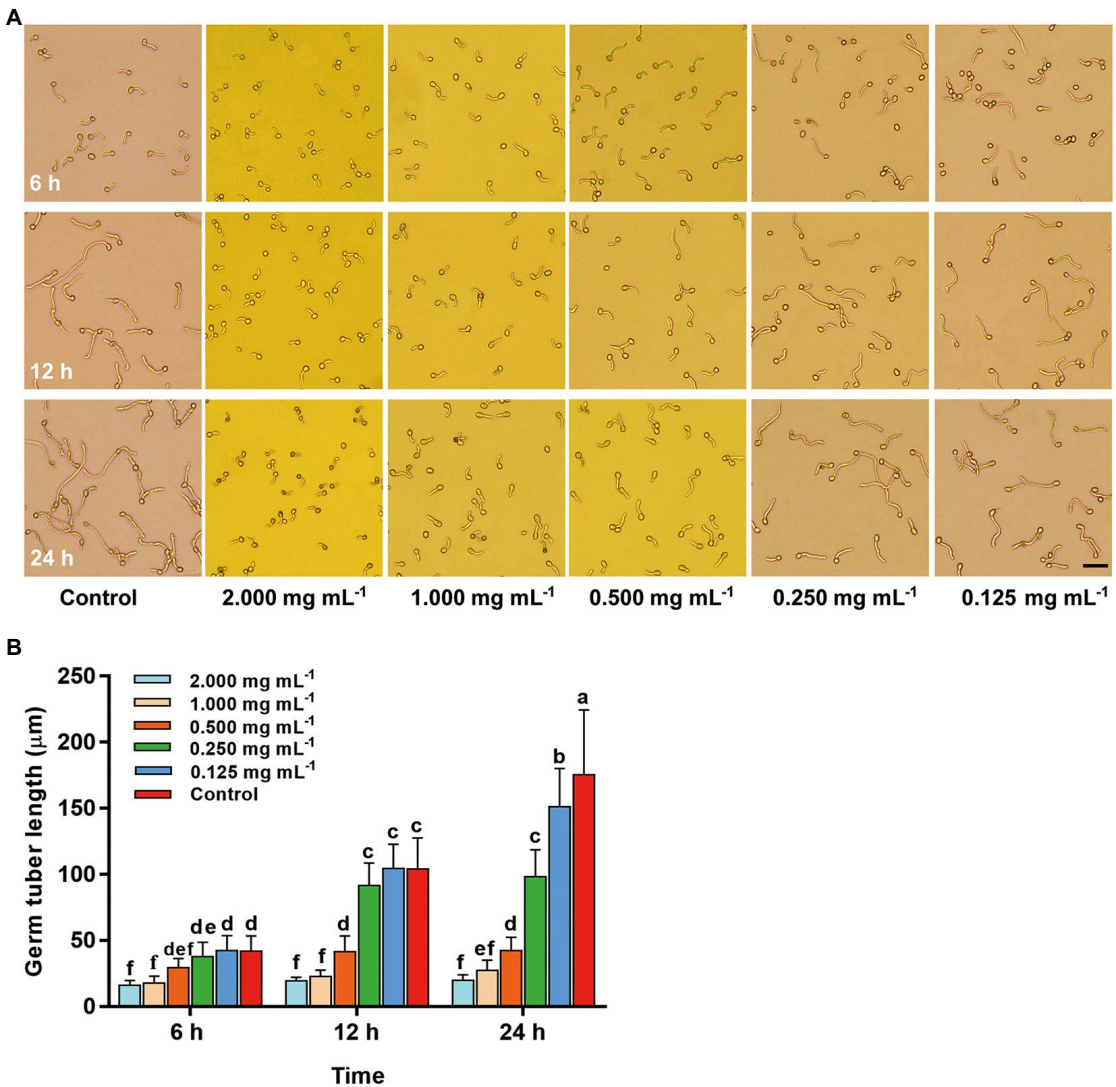
Changes of conidia and germ tubes of *B. cinerea* after treated with different concentrations of compound **2** in 24 h. **(A)** Images for morphological changes of conidia and germ tubes in different treatments Bar = 100 μm. **(B)** Inhibitory effect of compound **2** against germ tube elongation of *B. cinerea*. Columns with different letters represent significant difference at *p* < 0.05.

To further analyze the possible antifungal mechanism of compound **2**, the inhibitory effects against conidia germination and germ tube elongation of *B. cinerea* with different concentrations of compound **2** were tested. In Figure 8A, all concentrations of compound **2** had no effect on conidia germination. However, the inhibitory effect of compound **2** on germ tube elongation showed a dose-dependent relationship. At 12–24 h, in control, part of the conidia germinated at multiple ends, and germ tubes rapidly elongated, constricted, and formed septa, however, this phenomenon was not found in other treatments. The length of germ tube was significantly (*p* < 0.05) increased in control and treatment at the concentration of 0.125 mg mL^−1^ with time, and they were 175.52 μm and 151.40 μm, respectively, at 24 h (Figure 8B). The treatment at the concentrations of 2 mg mL^−1^ and 1 mg mL^−1^ had great inhibitory effect, and there were no significant changes in the length of germ tubes, which was only 16.07–27.38 μm within 24 h.

## Discussion

Insect symbionts are special microorganisms which live together with the host for a long time (Beemelmanns et al., 2016). In recent years, symbionts have been reported as an important source of new natural products which have diverse biological activities, novel molecular structures and complex biosynthetic pathways (Seabrooks and Hu, 2017; Shao et al., 2017; Van Arnam et al., 2018). Termite symbiosis is perhaps the longest-studied beneficial insect-microbe association (Scharf and Peterson, 2021). In general, the structure and environment of termite hindgut make contribute to the gut microbiota to live and thrive. These microbes include protozoa, bacteria and archaea, which can produce kinds of secondary metabolites (Ramadhar et al., 2014; Scharf, 2020). Therefore, termite symbiosis are valuable biological resources and have become one of the research hotspots. In our previous study, 44 actinomycetes isolates displayed antifungal activity against *Xylaria* sp. (Li J. et al., 2021). In this research, we focus on the antifungal activities for applications in agriculture. The strain sdu1201 was isolated from the hindgut of *O. formosanus*, and it was further designated as *Streptomyces* sp. sdu1201, according to 16S rRNA sequence and analysis of morphological (Figure 1).

So far, *Streptomyces* was reported to produce various kinds of secondary metabolites, especially antibacterial and antifungal compounds (Azish et al., 2021). In this study, the fermentation broth of *Streptomyces* sp. sdu1201 had antifungal activity against six phytopathogen fungi, among which the highest inhibition rate was to *B. cinerea* (Figure 3A). And its supernatant had a strong antagonistic ability against *B. cinerea* (Figure 3B), indicating that *Streptomyces* sp. 1201 may produce secondary metabolites with antifungal activity. Four compounds were isolated from *Streptomyces* sp. sdu1201 and identified as actinomycin Y6, actinomycin D, actinomycin X_0β_, and actinomycin G6 by data of HRMS and 1D NMR (Supplementary Figure 1; Supplementary Table 1; Figure 4). EC_50_ values are commonly used to evaluat drug potency (Li et al., 2015). In order to further determine which compound exhibit strong antifungal activity against *B. cinerea*, we determined the EC_50_ of four compounds. The results showed that actinomycin D (compound **2**) had the lowest EC_50_ (Table 1), which showed the strongest activity against *B. cinerea*.

Actinomycins are part of chromopeptide lactone antibiotics, among them, actinomycin D was reported to have significant anti-tumor, anti-virus, and anti-bacterial activities (Chen et al., 2012; Khieu et al., 2015). Actinomycin D is well-known to be used in medicine, but its application in agriculture is rarely reported. Zhao et al. first reported that actinomycin D can arrest primary root growth in *Arabidopsis* root by inhibiting ribosome biogenesis and reducing meristematic activity (Zhao et al., 2022). Another report pointed out that actinomycin D can caused mortality of second-stage juveniles and decreased egg hatching in *Meloidogyne incognita* with a dose dependent manner (Sharma et al., 2019). Actinomycin D had great antibacterial activity against *Ralstonia solanacearum* of tomato plants with a lower MIC (0.6 mg L^−1^) (Ling et al., 2020). It was also reported that actinomycin D showed strong antifungal activity against *Verticillium dahliae* by a membrane-splitting mechanism (Zeng et al., 2019). In addition, a number of biological activity tests showed that actinomycin D had a certain activity against *Fusarium graminearum*, *F. oxysporum*, *Rhizoctonia solani*, *Valsa mali*, *Magnaporthe grisea*, and *Dothiorella gregaria* (Khieu et al., 2015; Wei et al., 2017; Yin et al., 2019). In our study, the antifungal activity of actinomycin D against *B. cinerea* was firstly explored. It was demonstrated that actinomycin D had striking inhibition on *B. cinerea* and the control effect of actinomycin D on gray mold was also verified on strawberry fruits and leaves *in vitro* (Figures 6, 7), indicating that *Streptomyces* sp. sdu1201 and its secondary metabolites had potential uses as biocontrol agents.

To date, more than 30 strains were reported to be able to produce actinomycin D, but their yield was varied, such as *Streptomyces sindenensis* with 80 mg L^−1^ (Srivastava, 2008), *Streptomyces parvulus* DAUFPE 3124 with 133 mg L^−1^ (de Fátima et al., 2001), *Streptomyces griseoruber* with 210 mg L^−1^ (Praveen and Tripathi, 2009), *Streptomyces parvulus* Av-R5 with 360 mg L^−1^ (Chandrakar and Gupta, 2019), *Streptomyces flavogriseus* NJ-4 with 960 mg L^−1^ (Wei et al., 2017) and *Streptomyces* sp. MS449 with 1770 mg L^−1^ (Chen et al., 2012). Under nonoptimized conditions, the yield of actinomycin D of our *Streptomyces* sp. sdu1201 increased linearly from 1 to 7 d, and the highest was 51.1 mg L^−1^. Similarly, the dry weight of thallus was consistent with the trend of actinomycin D production (Supplementary Figure 6).

In recent years, many strategies for optimizing actinomycin D yield have also been reported. For example, screening different carbon and nitrogen sources (Praveen et al., 2007; Chandrakar and Gupta, 2019), adding amino acids such as L-asparagine or L-threonine (de Fátima et al., 2001), or breeding by UV mutagenesis (Srivastava, 2008), all could significantly increase the yield of actinomycin D. In addition, actinomycin D BGCs (Figure 5) and regulatory genes had been reported (Liu et al., 2019), which also can contribute to enhance the production of actinomycin D. Thus, we will improve the yield of actinomycin D from *Streptomyces* sp. sdu1201 in the future on account of its biocontrol potential.

*In vitro* bioassay on strawberry fruits, the fermentation broth of *Streptomyces* sp. sdu1201 plays an important role on biocontrol efficiency (Figure 6). Strawberry fruits in the treatment of S1201 were not wilted or affected, unified with those of in control, which revealed that the fermentation broth was harmless to strawberry fruits under this concentration. The same can be seen in bioassay on strawberry leaves with actinomycin D (Figure 7). Metabolites of *Streptomyces* sp. have been predicted to play an important role in biocontrol efficiency (Duan et al., 2020; Wei et al., 2020). After the leaves sprayed with actinomycin D under 62.5 mg L^−1^ for 4 d, there were still no visible changes, indicating that actinomycin D are safe to strawberry leaves under this concentration. The safety of actinomycin D on postharvest strawberry fruits and its application on strawberry plants in the field still need to be further studied.

The formation and germination of conidia of phytopathogenic fungi are very important in the gray mold disease cycle (Petrasch et al., 2019). Conidia germination and germ tube elongation, essential prerequisites for phytopathogenic fungi to effectively infect, have been in the limelight (Ortiz-Ribbing and Williams, 2006; An et al., 2016). Polarized growth of germ tubes was important for the pathogenicity of *B. cinerea* to hosts (Fischer et al., 2008; Zhao et al., 2021). Therefore, inhibiting conidia germination or growth arrest after germination was an effective method to control fungal diseases (Jarvis, 1994). In our study, actinomycin D could not restrain conidia germination of *B. cinerea*, but it effectively inhibited the polarized growth of germ tubes under the concentration of 0.25 ~ 2 mg mL^−1^ (Figure 8). In all treatments of actinomycin D, no conidia which germinated at multiple ends and formed septa were found, indicating that actinomycin D may delay the infection of *B. cinerea* to its host (Figure 8A). Besides, at 24 h, the conidia in 2 mg mL^−1^ treatment turned brown, which may decrease the cell viability (Xiao et al., 2021; Zhao et al., 2021). These two factors may be the main reasons for the smaller lesion area of strawberry fruits or leaves in the early stage of disease after actinomycin D treatment. In conclusion, this may be one of the mechanisms of actinomycin D against strawberry gray mold.

## Conclusion

In summary, our research shows that *Streptomyces* sp. sdu1201 isolated from the hindgut of the termite *O. formosanus* are able to produce actinomycin D, which can protect strawberry against *B. cinerea*. This study shed light on the important role of actinomycin D and *Streptomyces* sp. sdu1201 as biocontrol agents in the protection of strawberry against gray mold.

## Data availability statement

The datasets presented in this study can be found in online repositories. The names of the repository/repositories and accession number(s) can be found in the article/Supplementary material.

## Author contributions

DY, YL, KG, YY, and SZ performed the experiments. DY, RL, and YZ designed the experiments. DY and RL analyzed the data and wrote the manuscript. JN provided the strain *Streptomyces* sp. sdu1201. QD, CR, AL, JF, and JN contributed to preparing the final version of the paper. All authors contributed to the article and approved the submitted version.

## Funding

This work was supported by the National Key R&D Program of China (2019YFA0904000); the National Natural Science Foundation of China (81502962, 31970119, 32170038, 31670097, 32270088, and 32161133013); the 111 Project (B16030); the Shandong Provincial Natural Science Foundation of China (ZR2020MC015 and ZR2019ZD30); and the Fundamental Research Funds of Shandong University (2018GN021).

## Conflict of interest

DY, YY, and SZ were employed by Qingdao Zhongda Agritech Co., Ltd.

The remaining authors declare that the research was conducted in the absence of any commercial or financial relationships that could be construed as a potential conflict of interest.

## Publisher’s note

All claims expressed in this article are solely those of the authors and do not necessarily represent those of their affiliated organizations, or those of the publisher, the editors and the reviewers. Any product that may be evaluated in this article, or claim that may be made by its manufacturer, is not guaranteed or endorsed by the publisher.
