# Supplementary material for: Biocontrol of strawberry gray mold caused by Botrytis cinerea with the termite associated Streptomyces sp. sdu1201 and actinomycin D
Source: Front Microbiol. 2022 Nov 4;13:1051730. doi: 10.3389/fmicb.2022.1051730 (PMC9674021; doi:10.3389/fmicb.2022.1051730)
Supplement: Supplementary file 1 [file Data_Sheet_1.docx]

**Supplementary Material**

Biocontrol of Strawberry Gray Mold Caused by *Botrytis cinerea* with the Termite Associated *Streptomyces* sp. sdu1201and Actinomycin D

Daojing Yong^1,2^, Yue Li^1^, Kai Gong^1^, Yingying Yu^2^, Shuai Zhao^2^, Qiong Duan^1^, Cailing Ren^1^, Aiying Li^1^, Jun Fu^1^, Jinfeng Ni^1*^, Youming Zhang^1,3*^ and Ruijuan Li^1*^

^1^Helmholtz International Lab for Anti-Infectives, Shandong University-Helmholtz Institute of Biotechnology, State Key Laboratory of Microbial Technology, Shandong University, Qingdao, China

^2^Qingdao Zhongda Agritech Co., Ltd., Qingdao, China

^3^Chinese Academy of Sciences (CAS) Key Laboratory of Quantitative Engineering Biology, Shenzhen Institute of Synthetic Biology, Shenzhen Institute of Advanced Technology, Chinese Academy of Sciences, Shenzhen, China

*** Correspondence:**

Jinfeng Ni

jinfgni@sdu.edu.cn

Youming Zhang

zhangyouming@sdu.edu.cn

Ruijuan Li

liruijuan@sdu.edu.cn

**Table 1**. ^13^C NMR data of compounds **1**−**4**.

|  |  |  |  |  |  |  |  |  |  |  |
| --- | --- | --- | --- | --- | --- | --- | --- | --- | --- | --- |
| **Position** | **Pentapeptidolactone (*α*-ring, *δ*_C_)** | | | |  | **Position** | **Pentapeptidolactone (*β*-ring, *δ*_C_)** | | | |
|  | **1^a^** | **2^a^** | **3^b^** | **4^a^** |  |  | **1^a^** | **2^a^** | **3^b^** | **4^a^** |
| Thr 1 | 170.4 | 170.0 | 168.8 | 170.2 |  | HThr 1 | 168.1 | 170.2 | 169.1 | 171.0 |
| 2 | 56.1 | 56.4 | 55.5 | 54.3 |  | Thr 2 | 52.5 | 56.2 | 54.9 | 52.7 |
| 3 | 75.8 | 76.2 | 74.8 | 73.9 |  | 3 | 58.5 | 76.0 | 75.4 | 64.1 |
| 4 | 18.1 | 19.4 | 17.7 | 18.5 |  | 4 | 71.8 | 18.1 | 17.8 | 58.0 |
| Val 1 | 175.7 | 175.6 | 173.5 | 174.7 |  | Val 1 | 175.4 | 175.5 | 173.3 | 171.8 |
| 2 | 56.3 | 60.0 | 58.9 | 57.5 |  | 2 | 56.2 | 60.0 | 58.1 | 57.4 |
| 3 | 28.4 | 33.1 | 32.0 | 31.8 |  | 3 | 28.3 | 32.9 | 31.4 | 28.2 |
| 4 | 21.7 | 19.8 | 19.0 | 20.1 |  | 4 | 19.8 | 19.6 | 19.2 | 19.4 |
| 5 | 19.4 | 19.9 | 19.2 | 19.9 |  | 5 | 19.4 | 19.6 | 18.6 | 18.7 |
| HMPro/1 | 174.9 | 175.0 | 173.2 | 172.4 |  | OPro/1 | 175.2 | 175.4 | 173.2 | 175.6 |
| MPro 2 | 59.8 | 58.5 | 57.0 | 60.2 |  | MPro 2 | 54.0 | 58.3 | 56.6 | 56.5 |
| 3 | 75.9 | 32.3 | 31.3 | 76.4 |  | 3 | 39.3 | 32.1 | 31.4 | 26.9 |
| 4 | 35.5 | 23.9 | 23.0 | 35.6 |  | 4 | 210.1 | 23.9 | 70.1 | 21.8 |
| 5 | 52.6 | 49.6 | 47.6 | 51.4 |  | 5 | 49.6 | 49.6 | 54.8 | 39.5 |
| 6 | 19.3 |  |  | 19.3 |  |  |  |  |  |  |
| Sar 1 | 168.4 | 168.4 | 166.5 | 168.5 |  | Sar 1 | 168.4 | 168.6 | 166.5 | 172.0 |
| 2 | 42.9 | 52.5 | 51.4 | 48.4 |  | 2 | 39.4 | 52.5 | 51.5 | 40.3 |
| NMe | 33.0 | 35.6 | 35.1 | 32.9 |  | NMe | 32.2 | 35.5 | 35.1 | 35.4 |
| MeVal 1 | 169.9 | 169.9 | 166.7 | 169.2 |  | MeAla/1 | 170.3 | 168.8 | 167.7 | 173.6 |
| 2 | 72.1 | 72.0 | 71.4 | 72.1 |  | MeVal 2 | 58.3 | 72.0 | 71.4 | 49.6 |
| 3 | 24.0 | 28.3 | 27.1 | 23.8 |  | 3 | 15.0 | 28.3 | 27.2 | 15.0 |
| 4 | 21.8 | 23.8 | 21.8 | 20.6 |  | 4 |  | 23.8 | 21.8 |  |
| 5 | 19.5 | 21.7 | 19.4 | 19.4 |  | 5 |  | 21.7 | 19.4 |  |
| NMe | 35.4 | 39.3 | 39.5 | 35.4 |  | NMe | 33.2 | 39.3 | 39.4 | 31.4 |
|  |  |  |  |  |  |  |  |  |  |  |
| **Chromophore (*δ*_C_)** | |  |  |  |  |  |  |  |  |  |
| 1 | 102.9 | 103.0 | 102.8 | 99.0 |  | 8 | 126.8 | 126.4 | 126.3 | 126.3 |
| 2 | 149.0 | 149.0 | 147.1 | 152.1 |  | 9 | 133.4 | 133.8 | 131.4 | 129.4 |
| 3 | 180.4 | 180.5 | 179.2 | 180.3 |  | 9a | 129.3 | 130.6 | 129.6 | 133.0 |
| 4 | 114.3 | 114.2 | 113.7 | 114.9 |  | 10a | 146.5 | 147.1 | 146.1 | 148.0 |
| 4a | 147.2 | 146.5 | 145.1 | 147.6 |  | 11 | 17.3 | 15.0 | 166.2 | 18.1 |
| 5a | 141.8 | 141.8 | 140.8 | 141.9 |  | 12 | 7.6 | 7.6 | 7.9 | 7.9 |
| 6 | 130.6 | 129.1 | 128.5 | 130.2 |  | 13 | 169.9 | 168.1 | 15.3 | 168.5 |
| 7 | 131.4 | 131.3 | 130.5 | 131.3 |  | 14 | 168.2 | 170.3 | 173.0 | 168.1 |
| ^a^Measured in CD_3_OD. ^b^Measured in CDCl_3_. | | | | | | | | | | |

**Table 2.** Comparison of actinomycin D BGC from *Streptomyces* sp. sdu1201 with reference actinomycin D BGC from *S. anulatus* and *S. costaricanus* ZS0073

|  |  |  |  | |  |  |  | |  |
| --- | --- | --- | --- | --- | --- | --- | --- | --- | --- |
| **Gene** | **Size (bp/aa)** | **Putative function and annotation** | ***S. anulatus*  (HM038106.1)** | | |  | ***S. costaricanus* ZS0073  (MK234849.1)** | | |
|  |  |  | **Gene** | **Identity  (%)** | **Query Cover (%)** |  | **Gene** | **Identity  (%)** | **Query Cover (%)** |
| *GE004886* | 636/211 | hypothetical protein | *acmT* | 70.53 | 98 |  | *AcnU3* | 77.78 | 98 |
| *GE004885* | 564/187 | hypothetical protein | *acmS* | 73.08 | 97 |  | *AcnU4* | 83.96 | 100 |
| *GE004884* | 201/66 | mbtH-like protein | *acmR* | 77.27 | 100 |  | *AcnD* | 87.88 | 100 |
| *GE004883* | 237/78 | 4-MHA carrier protein | *acmD* | 67.53 | 98 |  | *AcnE* | 74.03 | 98 |
| *GE004882* | 1404/467 | Adenylation domain protein | *acmA* | 71.34 | 99 |  | *AcnN1* | 78.16 | 100 |
| *GE004881* | 7827/2608 | non-ribosomal peptide synthase | *acmB* | 68.74 | 100 |  | *AcnN2* | 73.93 | 100 |
| *GE004880* | 12717/4238 | non-ribosomal peptide synthetase | *acmC* | 74.65 | 99 |  | *AcnN3* | 78.30 | 99 |
| *GE004879* | 633/210 | hypothetical protein | *acmE* | 79.71 | 98 |  | *AcnF* | 86.06 | 99 |
| *GE004878* | 894/297 | aryl formamidase | *acmF* | 81.98 | 95 |  | *AcnG* | 83.28 | 96 |
| *GE004877* | 843/280 | tryptophan 2,3-dioxygenas | *acmG* | 74.47 | 100 |  | *AcnH* | 80.86 | 94 |
| *GE004876* | 1263/420 | kynureninase | *acmH/acmK* | 81.67/93.33 | 100/70 |  | *AcnL* | 85.71 | 100 |
| *GE004875* | 1041/346 | methyltransferase | *acmI/acmL* | 81.50/95.09 | 100/100 |  | *AcnM* | 87.57 | 100 |
| *GE004874* | 1296/432 | cytochrome P450 |  |  |  |  | *AcnP* | 83.95 | 93 |
| *GE004872* | 648/215 | LbmU-like protein | *acmJ/acmO* | 70.09/90.19 | 94/94 |  | *AcnO* | 77.31 | 94 |
| *GE004871* | 855/284 | TetR family transcriptional regulator | *acmU/acmP* | 64.46/88.38 | 95/100 |  | *AcnR* | 76.60 | 98 |
| *GE004870* | 885/294 | siderophore-interacting protein | *acmV/acmQ* | 74.20/90.48 | 96/100 |  | *AcnQ* | 76.53 | 99 |
| *GE004869* | 1077/358 | ABC transporter ATPase subunit | *acmW/acmrA* | 81.04/94.19 | 91/91 |  | *AcnT1* | 82.57 | 91 |
| *GE004868* | 768/255 | ABC 2-type transporter | *acmX/acmrB* | 83.53/96.08 | 100/100 |  | *AcnT2* | 87.84 | 100 |
| *GE004867* | 2298/765 | UrvA-like protein | *acmrC* | 93.91 | 98 |  | *AcnT3* | 85.05 | 97 |


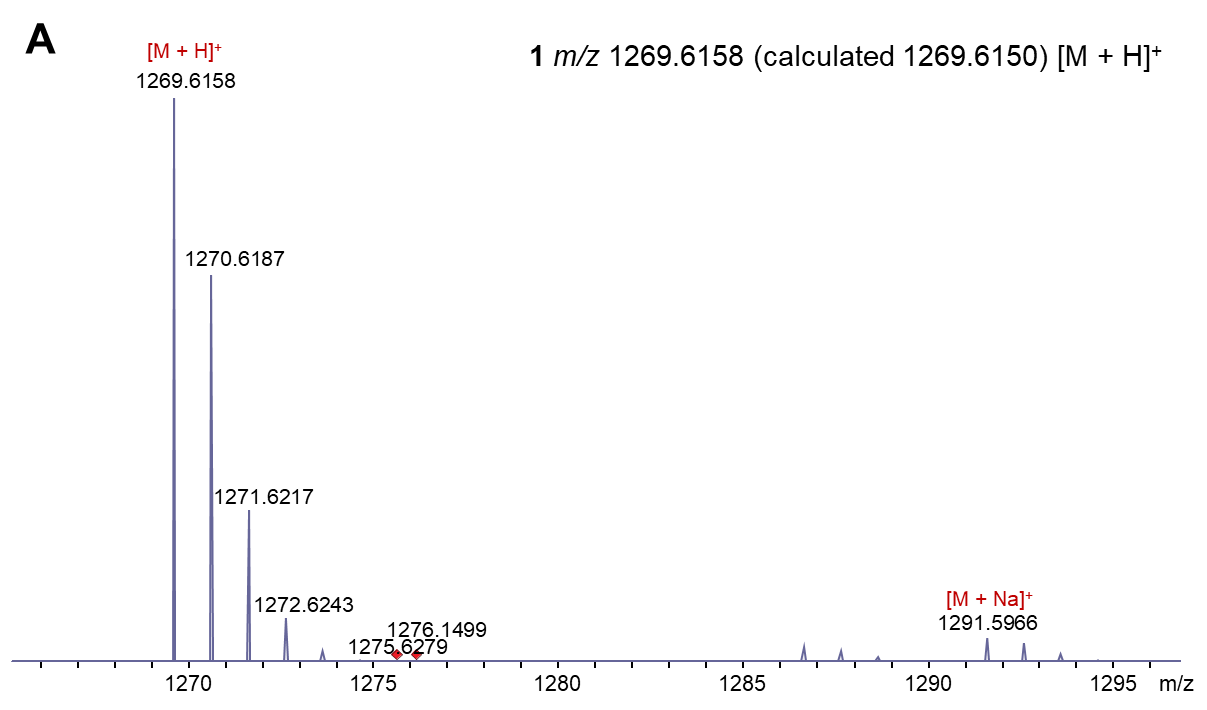

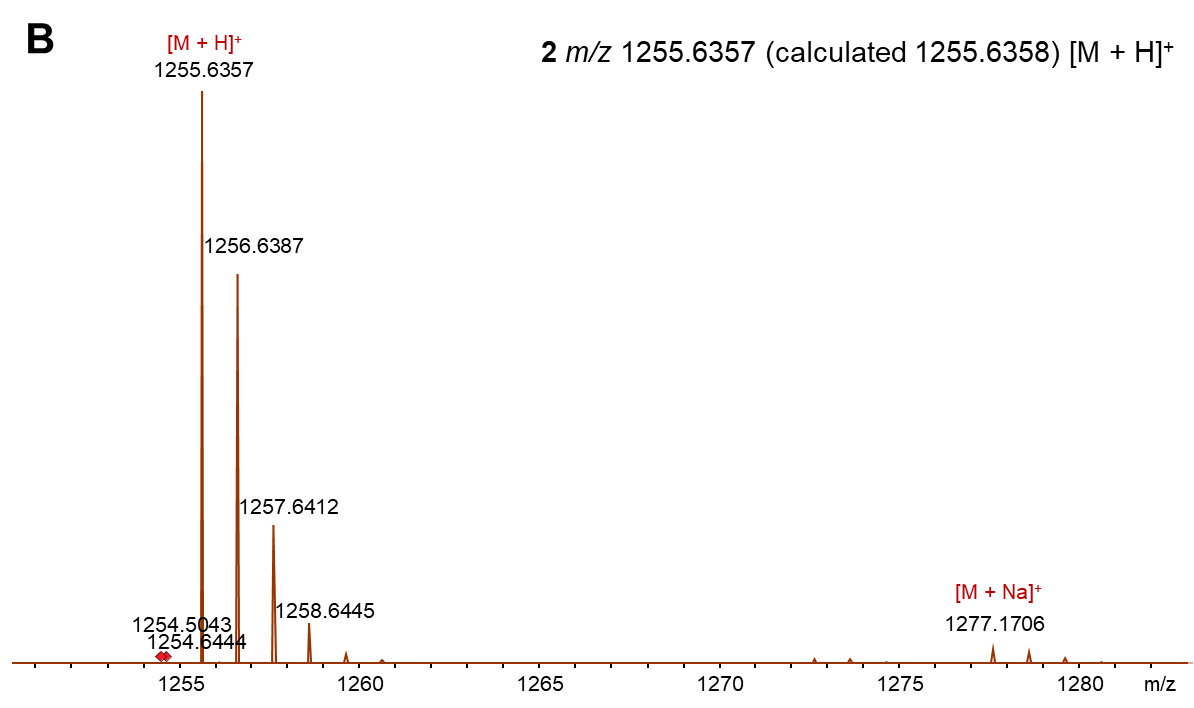


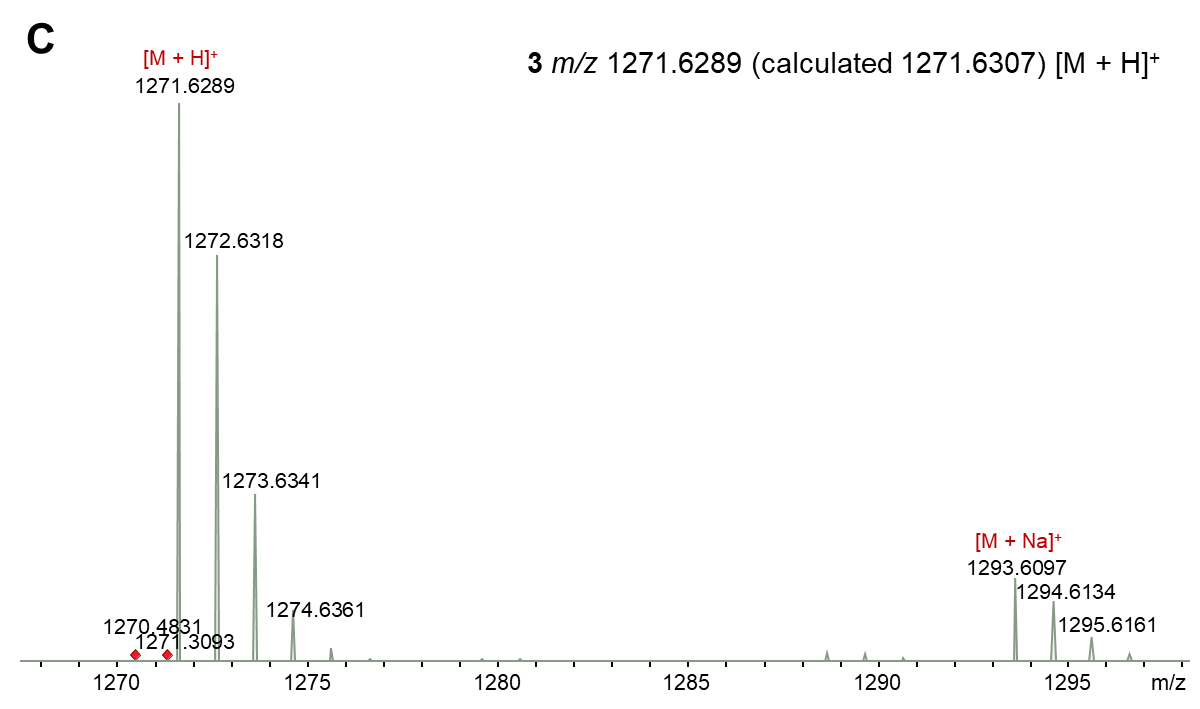

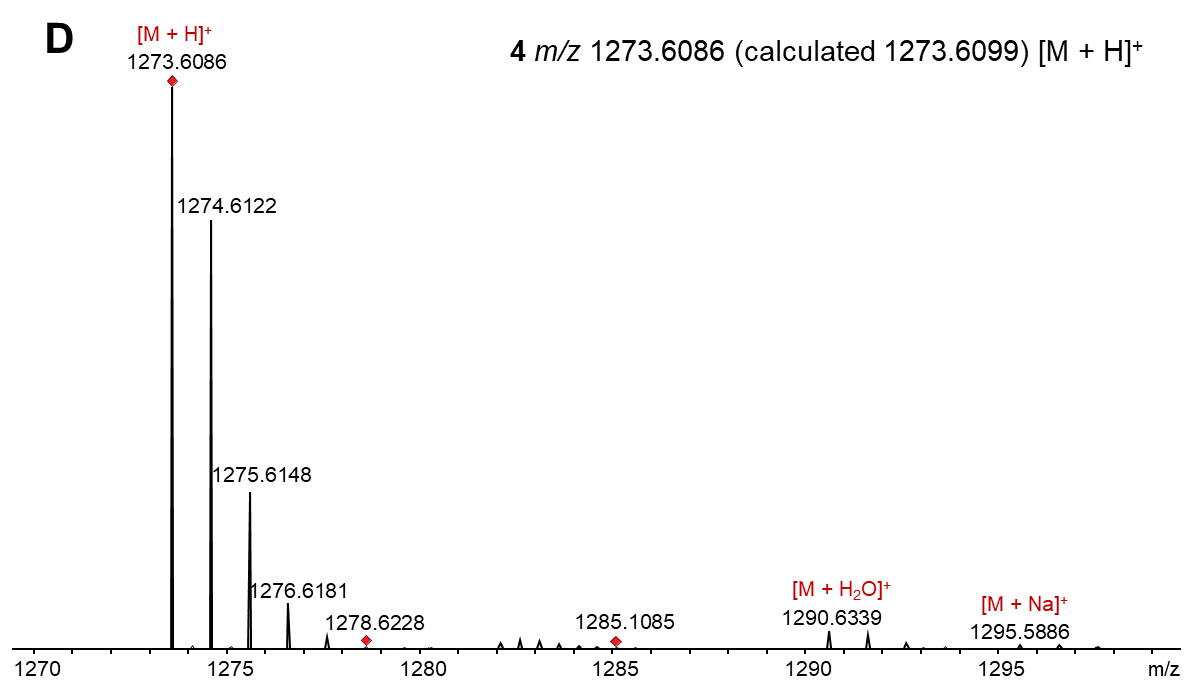


**Figure 1**. LC-HRMS data of four compounds. **(A)** Compound **1**. **(B)** Compound **2**. **(C)** Compound **3**. **(D)** Compound **4**.


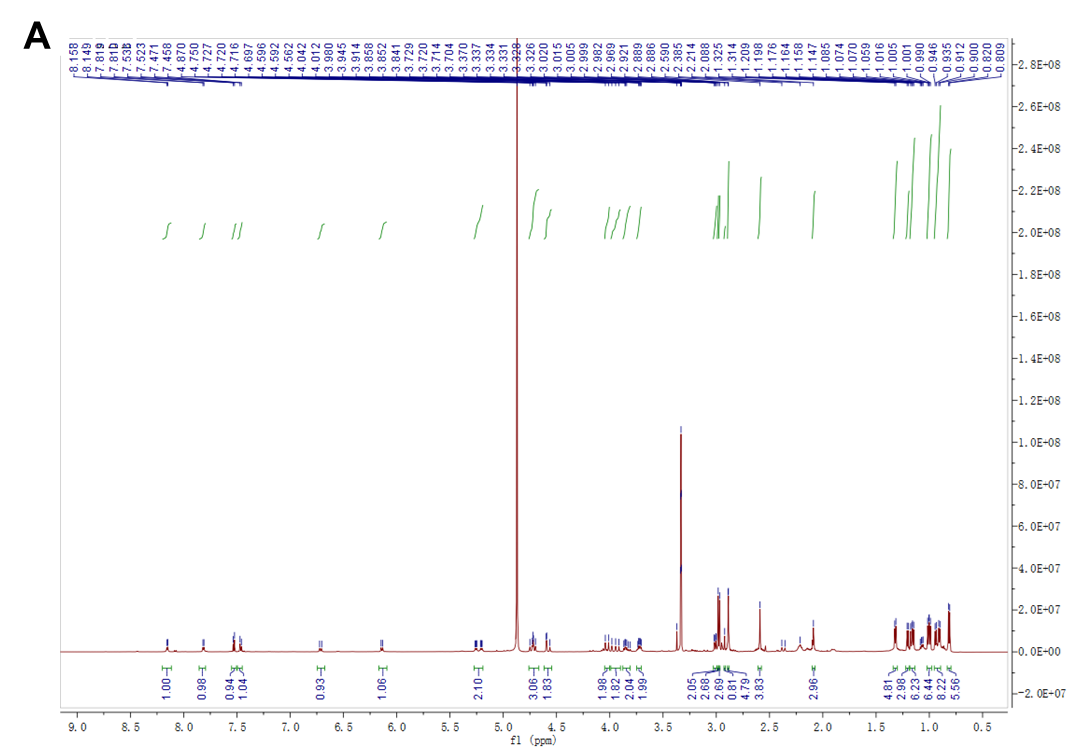


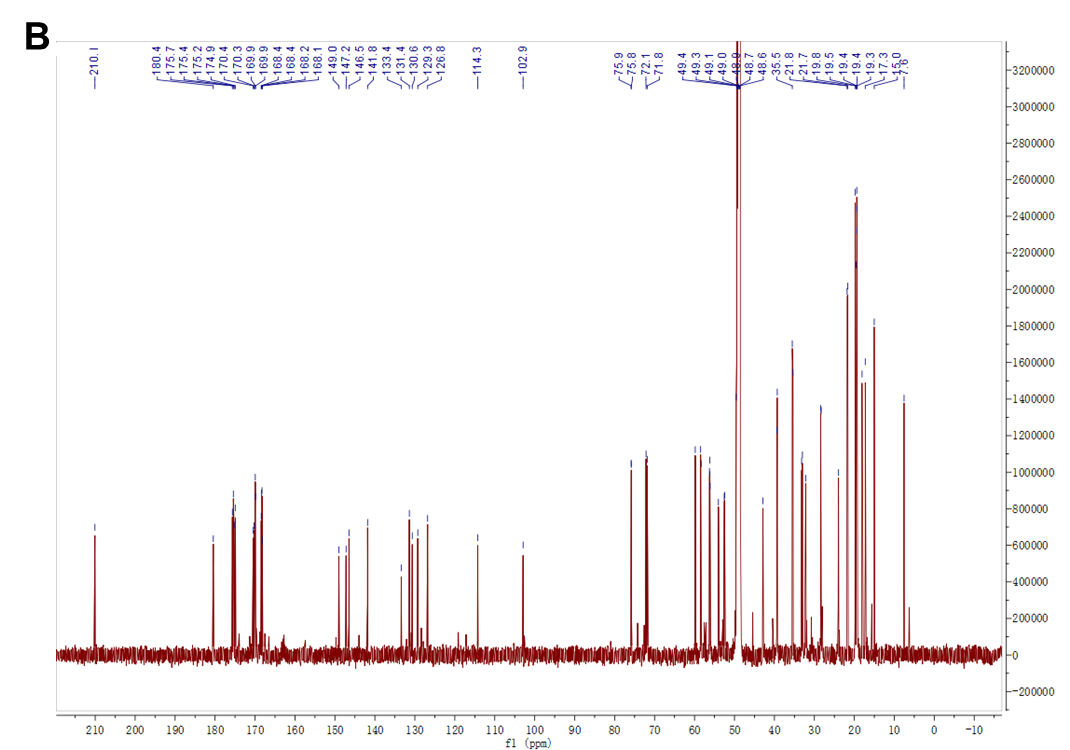


**Figure 2**. 1D NMR spectra of compounds **1** in CD_3_OD. **(A)** ^1^H NMR spectrum of **1**. **(B)** ^13^C NMR spectrum of **1**.


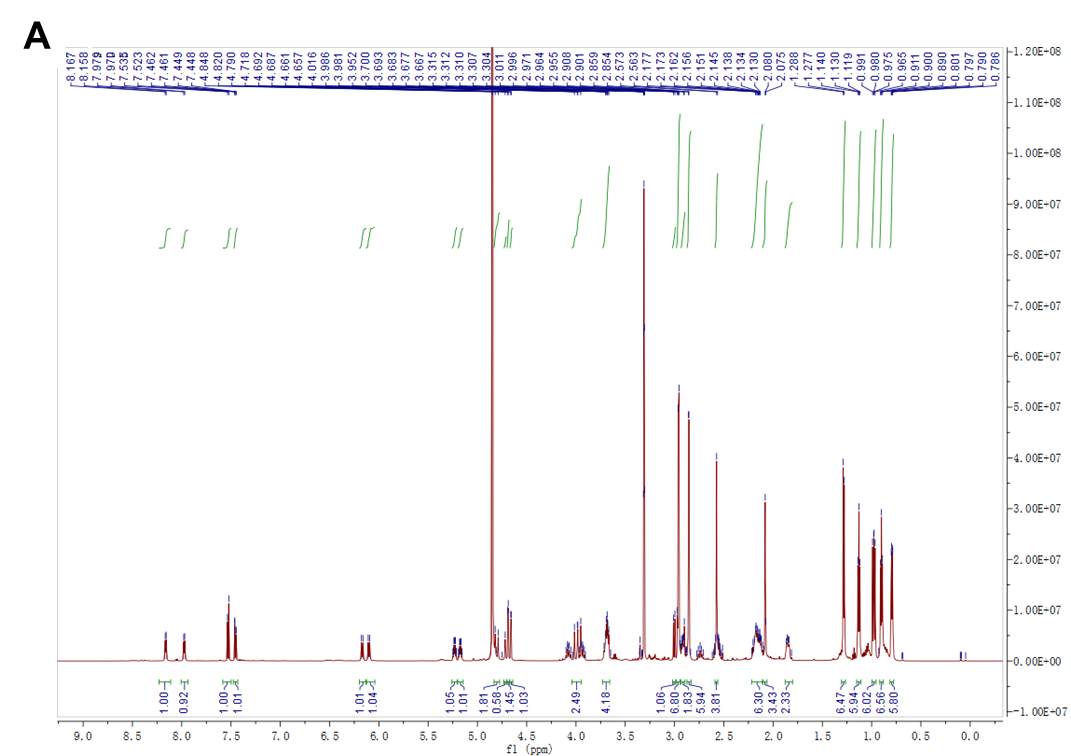


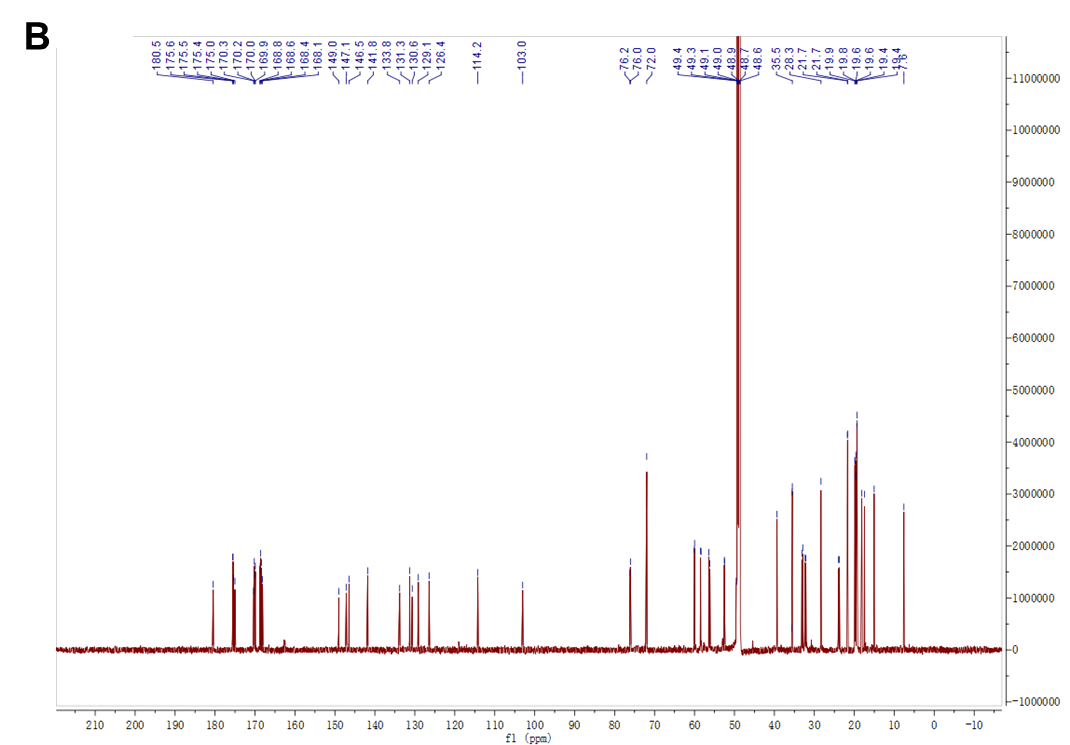


**Figure 3**. 1D NMR spectra of compounds **2** in CD_3_OD. **(A)** ^1^H NMR spectrum of **2**. **(B)** ^13^C NMR spectrum of **2**.


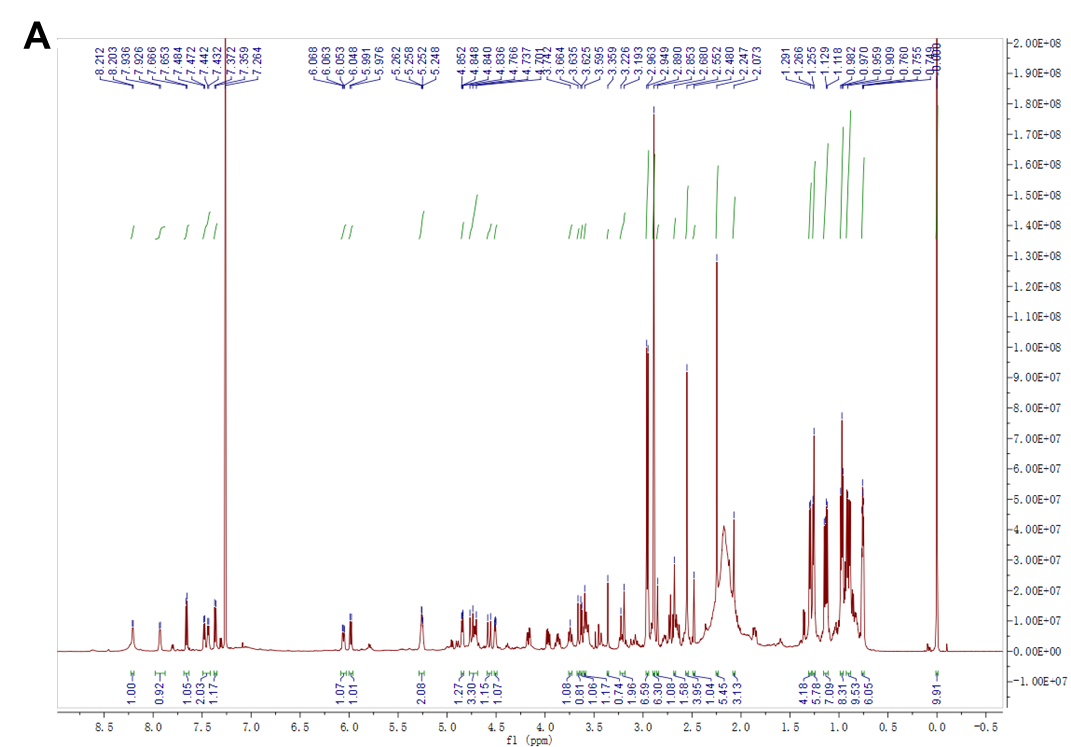


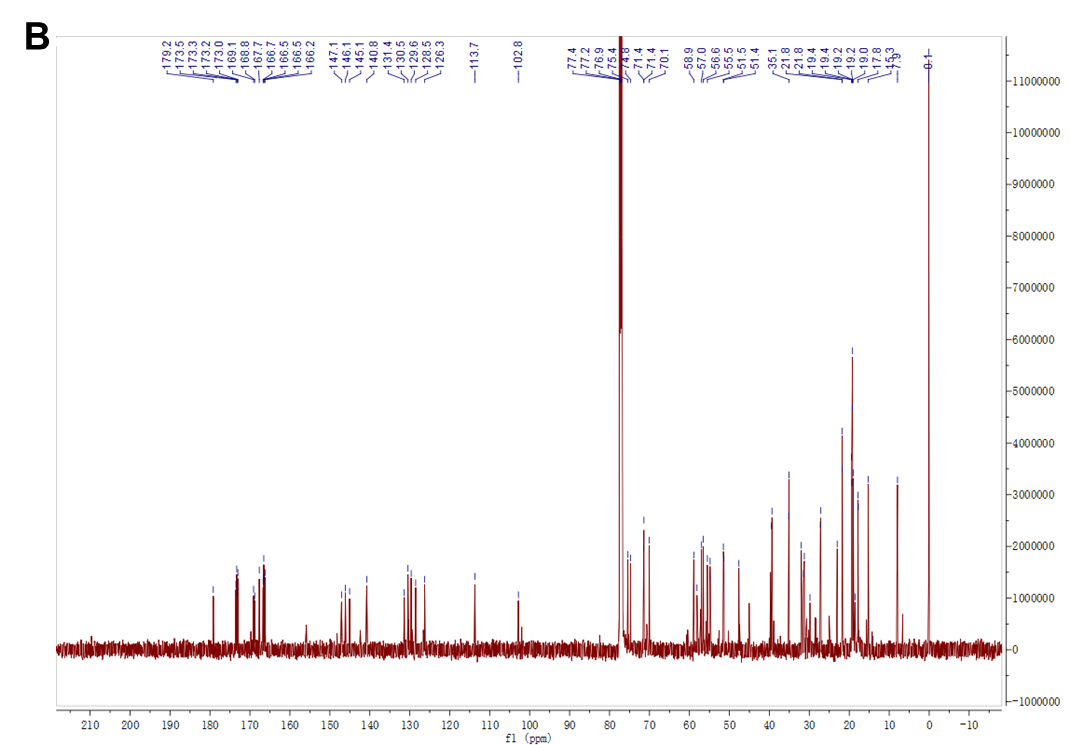


**Figure 4**. 1D NMR spectra of compounds **3** in CDCl_3_. **(A)** ^1^H NMR spectrum of **3**. **(B)** ^13^C NMR spectrum of **3**.


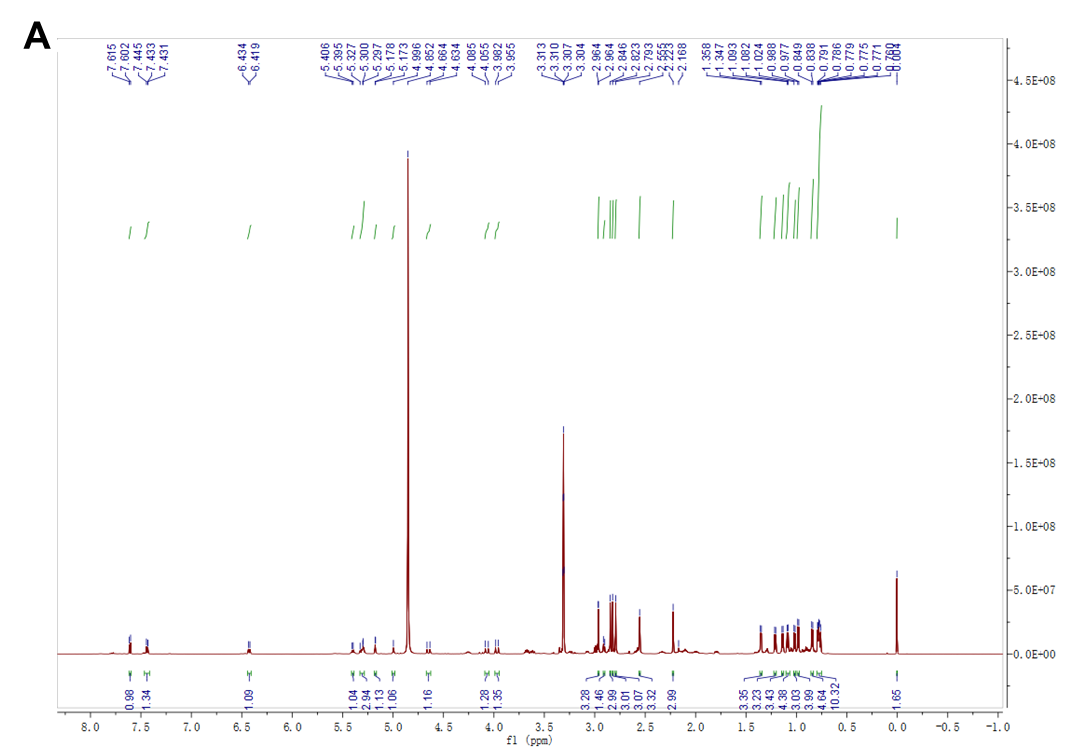


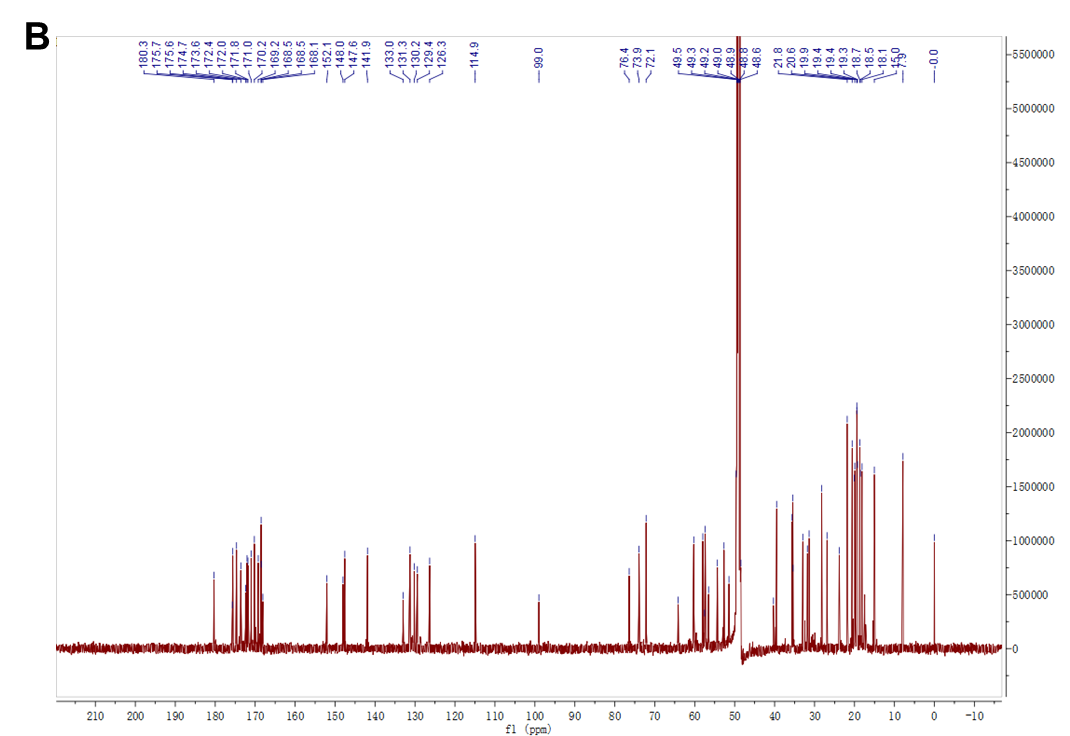


**Figure 5**. 1D NMR spectra of compounds **4** in CD_3_OD. **(A)** ^1^H NMR spectrum of **4**. **(B)** ^13^C NMR spectrum of **4**.

**Figure 6.** Production of actinomycin D from *Streptomyces* sp. sdu1201 in ISP-7 medium. **(A)** Content of actinomycin D and dry weight of thallus in fermentation broth of *Streptomyces* sp. sdu1201. **(B)** Standard curve of actinomycin D.
